# Combination strategies to maximize the benefits of cancer immunotherapy

**DOI:** 10.1186/s13045-021-01164-5

**Published:** 2021-09-27

**Authors:** Shaoming Zhu, Tian Zhang, Lei Zheng, Hongtao Liu, Wenru Song, Delong Liu, Zihai Li, Chong-xian Pan

**Affiliations:** 1 Chinese American Hematologist and Oncologist Network, New York, NY USA; 2 Department of Urology, Beijing Chao-Yang Hospital, Beijing, China; 3 Division of Medical Oncology, Department of Medicine, Duke Cancer Institute, Duke University, DUMC 103861, Durham, NC 27710 USA; 4 The Sydney Kimmel Comprehensive Cancer Center, Johns Hopkins University School of Medicine, Baltimore, MD 21287 USA; 5 University of Chicago, Chicago, IL USA; 6 Kira Pharmaceuticals, Cambridge, MA USA; 7 New York Medical College, Valhalla, NY USA; 8 Pelotonia Institute for Immuno-Oncology, The Ohio State University, Columbus, OH USA; 9 Harvard Medical School, West Roxbury, MA 02132 USA

**Keywords:** Immunotherapy, Immune checkpoint inhibitor, Cancer vaccine, Oncolytic virus, CAR-T, Cytokine

## Abstract

Immunotherapies such as immune checkpoint blockade (ICB) and adoptive cell therapy (ACT) have revolutionized cancer treatment, especially in patients whose disease was otherwise considered incurable. However, primary and secondary resistance to single agent immunotherapy often results in treatment failure, and only a minority of patients experience long-term benefits. This review article will discuss the relationship between cancer immune response and mechanisms of resistance to immunotherapy. It will also provide a comprehensive review on the latest clinical status of combination therapies (e.g., immunotherapy with chemotherapy, radiation therapy and targeted therapy), and discuss combination therapies approved by the US Food and Drug Administration. It will provide an overview of therapies targeting cytokines and other soluble immunoregulatory factors, ACT, virotherapy, innate immune modifiers and cancer vaccines, as well as combination therapies that exploit alternative immune targets and other therapeutic modalities. Finally, this review will include the stimulating insights from the 2020 China Immuno-Oncology Workshop co-organized by the Chinese American Hematologist and Oncologist Network (CAHON), the China National Medical Product Administration (NMPA) and Tsinghua University School of Medicine.

## Introduction

Recent major breakthroughs in cancer immunotherapy lie in the identification of immune checkpoints that cancer cells hijack to suppress anti-cancer immunity. With the approval of immune checkpoint blockers (ICBs) across cancer types, immunotherapy has revolutionized cancer treatment, especially with metastatic cancers where some patients, previously considered to be incurable, can enjoy long-term remission and survival. So far, the US Food and Drug Administration (FDA) approved ICBs include antibodies targeting programmed cell death 1 (PD1), PD1 ligand 1 (PD-L1) and cytotoxic T-lymphocyte-associated protein 4 (CTLA-4).

With FDA approvals of multiple ICBs across cancer types, new applications and approvals of cancer immunotherapy have stagnated. More recently, adoptive cell therapy (ACT), such as chimeric antigen receptor-engineered T (CAR-T) cells, has emerged as an effective therapy in hematological malignancies. While ICBs restore suppressed pre-existing anti-cancer immunity, CAR-T cells bypass antigen presentation, T cell priming and activation, thus directly attacking cancer cells. After administration, ACT is still governed by the downstream resistance mechanisms, especially those at the tumor microenvironment (TME). In addition to ICBs and ACT, novel strategies of immunotherapy are being explored to further improve the treatment efficacy and/or decrease immune-mediated toxicities.

**Fig. 1:**
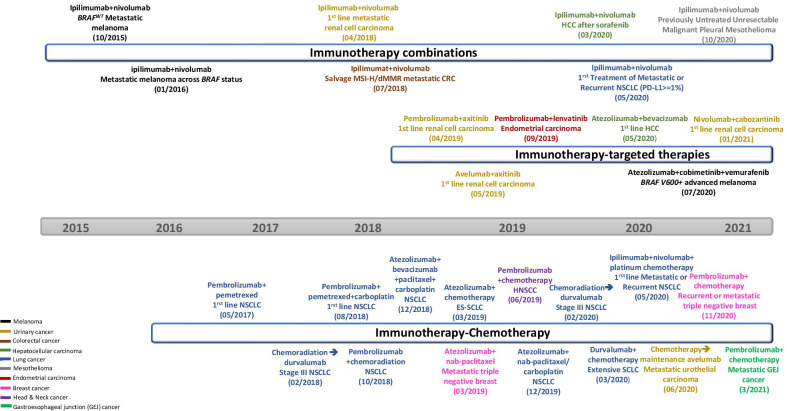
Timeline of the FDA approvals of combination therapy

Even though ACT is, in general, associated with high response rates, many patients eventually develop secondary resistance. On the other hand, the response rate of ICB monotherapies is usually around the 20% range across solid tumors. One strategy to improve cancer immunotherapy is to develop biomarkers, such as PD-L1, that can be used to select potential responders and/or exclude potential non-responders. Another strategy is to combine agents with different mechanisms of action and target multiple resistant mechanisms. So far, several combination therapies have already been approved by the FDA across different cancer types (Table 1 and Fig. 1). This review article will review emerging combination therapies, some of which were updated at the 2020 China Immuno-Oncology (IO) Workshop co-organized by the Chinese American Hematologist and Oncologist Network (CAHON), the China National Medical Product Administration (NMPA) and Tsinghua University [1, 2].

**Table 1:** Currently approved immunotherapy combinations in cancer

Combinations	Indications	Approval dates	References
Pembrolizumab + pemetrexed /platinum	First-line non-squamous NSCLC	May 10, 2017	[50, 296]
		August 21, 2018	
Chemoradiation followed by durvalumab	Stage III NSCLC	February 16, 2018	[86]
Chemotherapy and pembrolizumab	First-line NSCLC	October 30, 2018	[52]
Atezolizumab + bevacizumab, paclitaxel and carboplatin	First-line NSCLC	December 6, 2018	[297]
Atezolizumab + nab-paclitaxel/carboplatin	First-line Non-squamous NSCLC	December 3, 2019	[51]
Nivolumab + ipilimumab	First-line treatment of metastatic or recurrent NSCLC (PD-L1 > = 1%)	May 15, 2020	[298]
Nivolumab + ipilimumab + 2 cycles of Pt chemo	First-line treatment of metastatic or recurrent NSCLC	May 26, 2020	[299]
Atezolizumab + etoposide/carboplatin	ES-SCLC	March 18, 2019	[53]
Durvalumab + chemo	Extensive SCLC	March 30, 2020	[54]
Nivolumab + ipilimumab	First-line advanced RCC	April 16, 2018	[300]
Axitinib + pembrolizumab	First-line advanced RCC	April 22, 2019	[301]
Avelumab plus axitinib	First-line advanced RCC	May 14, 2019	[302]
Nivolumab + cabozantinib	First-line advanced RCC	January 22, 2021	[303]
Chemotherapy, trastuzumab and pembrolizumab	Advanced unresectable or metastatic HER2-positive gastric or gastroesophageal junction adenocarcinoma	May 5, 2021	[304]
Chemotherapy + pembrolizumab	Locally advanced or metastatic gastric or gastroesophageal junction adenocarcinoma	March 23, 2021	[305]
Atezolizumab + nabpaclitaxel	Metastatic triple negative breast	March 8, 2019	[55]
Pembrolizumab + chemotherapy	Recurrent or metastatic triple negative breast	November 13, 2020	[56]
Pembrolizumab + chemotherapy	HNSCC	June 11, 2019	[57]
Pembrolizumab + lenvatinib	Endometrial carcinoma	September 17, 2019	[306]
Nivolumab + ipilimumab	Previously untreated unresectable malignant pleural mesothelioma	October 2, 2020	[307]
Nivolumab + ipilimumab	Hepatocellular carcinoma after Sorafenib	March 11, 2020	[308]
Atezolizumab + bevacizumab	HCC 1st-line	May 29, 2020	[309]
Nivolumab + ipilimumab	Salvage MSI-H/dMMR metastatic CRC	July 11, 2018	[310]
Nivolumab + ipilimumab	BRAF^WT^ Metastatic melanoma	October 1, 2015	[311]
Nivolumab + ipilimumab	Metastatic melanoma across BRAF status	January 23, 2016	[312]
Atezolizumab + cobimetinib and vemurafenib	BRAF V600 + advanced melanoma	July 30, 2020	[313]
Chemotherapy followed by avelumab	Locally advanced or metastatic urothelial carcinoma	June 30, 2020	[58]

## Cancer-immunity cycle

**Fig. 2:**
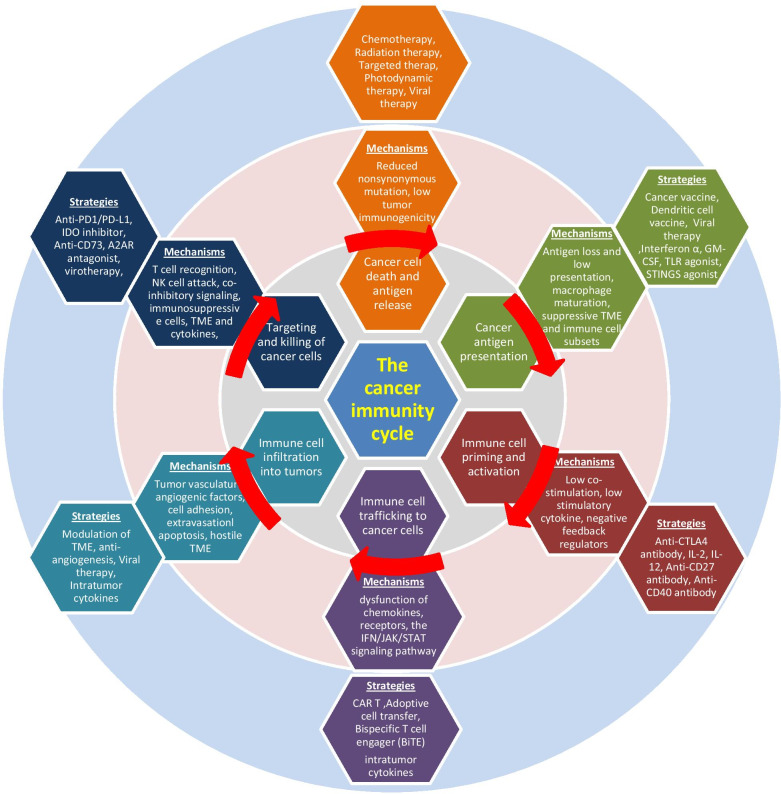
The cancer-immunity cycle, resistant mechanisms and potential solutions

In 2013, Chen and Mellman (2013) used the concept of “the Cancer-Immunity Cycle,” which dissects the anti-cancer immune response process similar to the way the body mounts response toward any foreign antigens (Table 2 and Fig. 2) [3]. The cycle starts with cross-presentation of cancer-associated antigens from cancer cells to the major histocompatibility complex (MHC) molecules on the antigen presenting cells (APCs). Cancer antigens encompass cancer neoantigens from genomic alterations (mutations, translocations, readthrough and frame shifts), cancer associated proteins normally expressed at immune privileged sites, viral proteins and others (Step 1). APCs, upon capturing of cancer antigens, migrate to secondary lymphoid organs (Step 2). These APCs prime and activate naïve T cells via MHC-antigen-T cell receptor (TCR) interaction, along with a hierarchy of costimulatory signals, such as the CD28/B7-1/2-mediated signaling (Step 3). Activated immune cells then enter the circulation system (step 4), infiltrate into the tumor microenvironment (Step 5), recognize tumor cells through the interaction of the TCR and its cognate antigen presented on MHC of tumor cells (Step 6) and kill their target cancer cells (Step 7). After killing the targeted cancer cells, release of more tumor antigens further fuels the anti-cancer immunity cycle.

**Table 2:**
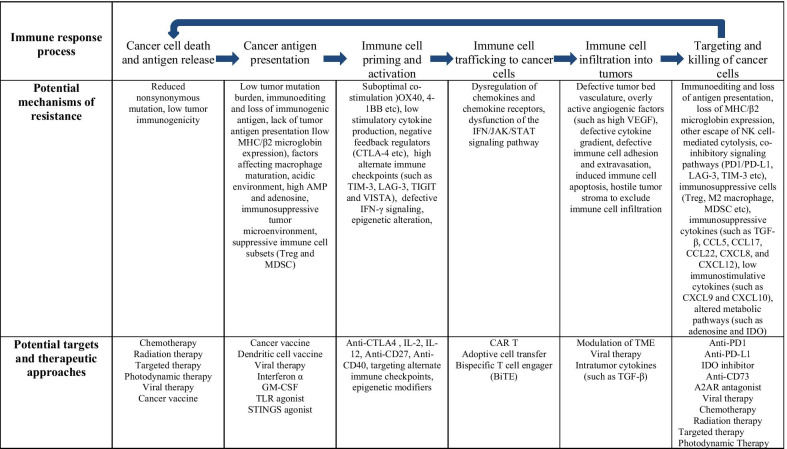
The cancer-immunity cycle, resistant mechanisms and potential solutions

## Resistant mechanisms along the cancer-immunity cycle

Cancer cells have been found to have intrinsic mechanisms bypassing every possible step along the cancer-immunity cycle to evade anti-cancer immunity (Table 2 and Fig. 2). At the initiation of the anti-cancer immune response, some cancers with low tumor mutation burden or low immune cell infiltration (such as in prostate cancer) may not elicit sufficient immune responses. Loss of MHC expression, loss or mutation of β2-microglobulin and mutations within the TCR binding domain of MHC have all been associated with escape from anti-cancer immunity [4–7].

CTLA4 is the first target of ICBs approved by the FDA [8]. In addition to CTLA4, several other negative regulators such as T-cell immunoglobulin, mucin domain-3 protein (TIM-3), lymphocyte-activation gene 3 (LAG-3), T-cell immunoreceptor tyrosine-based inhibition motif domain (TIGIT) and V-domain immunoglobulin-containing suppressor of T-cell activation (VISTA) [9–13], have been identified and are currently being tested in clinical trials to determine their potential as targets for cancer immunotherapy. Other than negative regulators, suboptimal co-stimulation molecule expression, inefficient cytokine production and heightened infiltration of immunosuppressive immune cells have all been found to contribute to weakened anti-cancer immunity.

After immune cell priming and activation, any defects affecting immune cell trafficking, migration and infiltration into the tumor microenvironment can invalidate anti-cancer immunity. Vascular endothelial growth factor (VEGF) plays important roles in angiogenesis as well as multiple facets of anti-cancer immunity. It decreases trafficking and extravasation of cytotoxic T cells, promotes infiltration of T_reg_ cells into the tumor bed [14] and enhances the expression of PD-1 and other inhibitory checkpoints involved in CD8^+^T cell exhaustion [15]. In mouse models, VEGF also impedes the commitment and progression of lymphoid progenitors to the T-cell lineage [16].

Cytokines within the TME not only affect immune cell migration and recruitment to the tumor site, but also modulate immune cell activities. Some cytokines, such as Chemokine (C-X-C motif) ligand 9 (CXCL9), CXCL10 and CXCL11, elicit chemotactic function and attract cytotoxic T cells while other cytokines, as seen with CCL5, CCL17, CCL22 and CXCL8, attract myeloid-derived suppressor cells (MDSCs) and T_reg_ cells contributing to the immunosuppressive TME [17–19]. In addition to cytokines, transforming growth factor beta (TGF-β) is a multipotent growth factor that affects cell growth and differentiation, apoptosis and immunosuppression. It is present in high concentrations in the TME because of production by cancer, stromal and immune cells. In general, it inhibits anti-cancer immunity through inhibiting the function of effector immune cells and promoting suppressive cells [20]. Both cytokines and TGF-β have already been experimentally targeted for cancer immunotherapy.

Once immune cells enter the TME, numerous mechanisms have been identified to elicit resistance to anti-cancer immunity, including cancer cell intrinsic factors, immune cells and the immunosuppressive milieu. As discussed above, through immunoediting and selection pressure from anti-cancer immunity, cancer cells with loss or decrease of antigen presentation can survive anti-cancer immunity and proliferate to become resistant cancers. Upregulation of immunosuppressive signaling pathways, such as PD-1, PD-L1, LAG-3 and TIM-3, infiltration of immunosuppressive cells, such as T_reg_ cells, MDSC, M2 macrophages, a hypoxic and acidic environment, or metabolic alterations in the tumor microenvironment, have all been found to negatively contribute to anti-cancer immunity.

Currently, the FDA-approved ICBs target the immune cell priming and activation (anti-CTLA4 antibody) or the final negative regulation of T effector cells (anti PD-1 and anti-PD-L1 antibodies). As these inhibitors only affect one to two steps of the anti-cancer immunity pathway, it is not surprising that only a minority (around 20%) of patients achieve cancer response with single agents. Slightly higher response rates have been observed with anti-CTLA4 and anti-PD1/PD-L1 combination treatments, at the cost of higher immune-mediated toxicities. Combination therapies are currently being extensively explored to target multiple defects along the immunity cycle and cancer intrinsic alterations and improve the anti-cancer efficacy, which will be covered in the following sections.

## Combinations of chemotherapy and immunotherapy

Most chemotherapeutic agents were developed through its direct cytotoxic effects without consideration of the effects on immune system. The interplay between chemotherapy and immunotherapy has been demonstrated in mouse models where mice with intact immune systems had significantly improved tumor responses to anthracyclines [21]. To date, multiple studies have demonstrated the contribution of cytotoxic chemotherapy to anti-cancer immunity, leading to several FDA-approved combination therapies with immunotherapy (Table 2) [22].

### Mechanisms of action

#### Debulking of tumors

One of the major benefits achieved by cytotoxic chemotherapy is tumor debulking. Tumor cells are the major contributor to immunosuppressive TME. Hence, reduction of cancer cell mass decreases production of immunosuppressive factors. Furthermore, reduction of tumor cell mass decreases the volume of cancer cells needed to be eliminated by immune cells. This can have dramatic consequences, especially in those tumors with limited immune cell infiltration at TME.

#### Immunogenic cell death (ICD)

ICD is a form of regulated cell death that is amenable to activating the adaptive immune response in immunocompetent hosts [23]. Numerous studies have shown that cytotoxic chemotherapy induces ICD and potentiates immunotherapy [24]. Insult of cancer cells by cytotoxic chemotherapy leads to release and relocation of damage-associated molecular patterns (DAMPs) that increase the adjuvanticity of cancer cells [25]. Release of intracellular molecules, such as ATP, enhances the recruitment of APCs; cytoplasmic annexin A1 released from cancer cells interacts with formyl peptide receptor 1 to promote interaction of dendritic cells and damaged cancer cells; exposure of endoplasmic reticulum chaperone proteins, such as heat shock protein 70 (HSP70), HSP90 and calreticulin, promotes the phagocytosis of stressed cancer cells by dendritic cells; cytosolic DNA and RNA stimulate the secretion of type I interferon and other proinflammatory cytokines through the cyclic GMP-AMP synthase (cGAS)/stimulator of interferon genes (STING) pathway, toll-like receptor 3 (TLR3) and TLR9; Type I interferon and other molecules released by stressed cancer cells, such as high mobility group box 1 (HMGB1), promote dendritic cell maturation and antigen presentation to T cells; and C–C motif chemokine ligand 2 (CCL2), C-X-C motif chemokine ligand 1 (CXCL1) and CXCL10 facilitate T-cell recruitment.

#### Increase in antigenicity of cancer cells

While ample evidence exists that chemotherapy increases the adjuvanticity of cancer cells through ICD, less is known about enhancement of antigenicity by chemotherapy. Many of the commonly used cytotoxic agents, such as anthracyclines, cyclophosphamide, platinum and taxanes, target cell cycle progression in proliferating cells and induce apoptosis. After tumor cell death, antigen-presenting cells engulf dying tumor cells and present tumor neoantigens to immune cells.

In addition, several other studies show that cytotoxic agents upregulate antigen-presenting machinery. Gemcitabine can significantly upregulate the expression of human leukocyte antigen (HLA)-A, B and C through increased expression of β2-microglobulin and alter the peptide antigen repertoire expressed on HLA class I [26]. A similar phenomenon is also observed with topotecan which upregulates HLA class I expression through activation of NF-κB/Interferon-β/MHC-I signaling axis [27]. As discussed above, ICD and stimulation of the cGAS/STING pathway induces type I interferon production which can upregulate HLA class I molecule expression and antigen presentation.

#### Depletion of immunosuppressive cells

Several subpopulations of immune cells are known to suppress anti-cancer immunity. Cytotoxic chemotherapy, such as platinum, cyclophosphamide, gemcitabine and 5-fluorouracil, can clearly reduce MDSCs in both humans and mice [28–31]. Trabectedin selectively depletes monocytes/macrophages through activation of caspase-8-dependent apoptosis [32]. Human T_reg_ cells lack the expression of cyclophosphamide-excreting transporter ABCB1 and are more sensitive to cyclophosphamide treatment than other immune cells [33]. Furthermore, chemotherapy alters the TME and favors the differentiation of immune cells supporting anti-cancer immunity. For example, cyclophosphamide and doxorubicin favor the M1 differentiation of tumor-associated macrophages [34].

#### Modulation of gene expression

In addition to the cytotoxic chemotherapy, another major class of small molecular drugs are epigenetic modulators. Epigenetic modulation, such as DNA methylation, histone modification, chromatin remodeling and the readout of these modifications, has tremendous impact during oncogenesis and is a critical event in some cancers, such as loss of tumor suppressor genes from DNA methylation. Hence, epigenetic modulators constitute an ever-expanding class of anti-neoplasm agents.

In addition to direct induction of ICD and stimulation of antitumor immunity, as seen with histone deacetylase (HDAC) inhibitors vorinostat and panobinostat [35], another major contributing mechanism to the synergy between epigenetic modulators and immunotherapy is through gene expression modification. Both HDAC and DNA methyltransferase (DNMT) inhibitors have been shown to upregulate the antigen processing and presentation machinery. Both HLA class molecules [36, 37] and tumor-associated antigens [38] have been found to be upregulated by epigenetic modulators. Epigenetic modulators also have direct impacts on the immune system to potentiate anti-cancer immunity. They can upregulate co-stimulatory molecules, such as CD80, CD86 and ICAM-1, and immune checkpoints CTLA4, PD1 and PD-L1 [39]. Furthermore, cytokines can also be induced, and response to immunotherapy can be augmented by epigenetic modulators [40]. The innate immune system can be modified by epigenetic modulators as well. Activating receptor NKG2D on the surface of NK cells and stressing-inducing ligand MICA and MICB on tumor cells can all be induced by HDAC inhibitors to increase NK cell killing of tumor cells [41, 42].

#### Potentiation and restoration of sensitivity to chemotherapy

Several studies showed that potentiation of immunotherapy and cytotoxic chemotherapy is reciprocal. Some patients with chemoresistant tumors responded to chemotherapy re-challenge upon disease progression on anti-PD1 therapy. In both Hodgkin’s lymphoma and non-small cell lung cancer, increased response to salvage chemotherapy was observed after disease progression on immune checkpoint blockade [43, 44].

#### Detrimental effects of chemotherapy on immunotherapy

One of the major detrimental effects of chemotherapy to the immune system is lymphodepletion which can be immunosuppressive. In fact, some of the immunosuppressive drugs used in clinic to treat autoimmune diseases are cytotoxic chemotherapy used for cancer treatment, but with different doses and schedules. It is still controversial whether lymphodepletion induced by chemotherapy is suppressive for anti-cancer immunity. Lymphodepletion associated with cancer chemotherapy is usually associated with rebound of lymphocyte counts and an immune system “reset.” One study showed the uneven recovery of different immune cell subpopulations tilting to anti-cancer immunity [45].

Chemotherapy can also affect tertiary lymphoid structures (TLS) [46, 47]. TLS are ectopic lymphoid organizations developed in non-lymphoid tissues, including cancer, and display similar organization as secondary lymphoid organs, such as lymph nodes. Extensive data suggest that TLS function similarly to lymph nodes in recruiting lymphocytes into tumors, and mounting local and systemic immune response against cancers. Overall, the presence and high densities of TLS in tumors favorably correlate with prognosis in multiple cancer types, and sometimes independent of the pathological TNM (tumor-lymph node-metastasis) staging [46–48]. The lymphodepleting effect of chemotherapy can also affect TLS either by the direct cytotoxic effect of chemotherapeutic drugs or associated therapies, such as corticosteroids [49].

### FDA-approved chemoimmunotherapy combinations

Many clinical trials with combinations of chemoimmunotherapy have been conducted in almost all major cancers with several FDA approvals (Fig. 1, Tables 1 and 3). The poster child of the combinations can be found in lung cancer. In the Keynote-189 trial with 616 lung adenocarcinoma patients, pembrolizumab with platinum-based doublet chemotherapy significantly improved the overall survival (OS) when compared to chemotherapy alone (HR 0.49, 95% CI 0.38–0.64, *p* < 0.001) [50]. While the benefit was greatest in patients whose tumors had PD-L1 > 50% expression, OS was improved across all patient subsets regardless of the PD-L1 status. In the Impower 130 clinical trial, anti-PD-L1 antibody atezolizumab was combined with carboplatin and nab-paclitaxel as it does not require corticosteroid. The combination group was associated with prolonged OS of 18.6 versus 13.9 months (HR 0.79, 95% CI 0·64–0·98, *p* = 0·033) [51]. Similar survival benefits were observed in metastatic lung squamous cell carcinoma, where pembrolizumab combined with carboplatin-doublet chemotherapy significantly improved OS (15.9 versus 11.3 months; HR 0.64, 95% CI 0.49–0.85, *p* < 0.001) [52].

**Table 3:** FDA-approved chemotherapy and immunotherapy combination

Cancer	Line of therapy	Chemotherapy	Immunotherapy	Clinical benefit	Statistics	Trial name and reference
NSCLC-non-squamous	Metastatic, first-line	Pemetrexed + platinum	Pembrolizumab	OS at 12 Mos: 69.2% versus 49.4%	HR 0.49; 95% CI 0.38–0.64; *P* < 0.001	KEYNOTE**-**189 [50],
NSCLC-non-squamous	Metastatic, first-line	Carboplatin + nabpaclitaxel	Atezolizumab	OS: 18.6 versus 13.9 Mos	HR 0·79; 95% CI 0·64–0·98; *p* = 0·033	IMpower 130, [51]
NSCLC-non-squamous	Metastatic, first-line	Carboplatin + paclitaxel + bevacizumab	Atezolizumab	OS: 19.2 versus 14.7 mo	HR 0.78; 95% CI 0.64 to 0.96; *P* = 0.02	IMpower 150, [297]
NSCLC	Metastatic, first-line	Platinum doublet	Nivolumab + ipilimumab	OS 15.6 versus 10.9 m;	HR 0.66; 95% CI 0.55–0.80; *P* = 0.00065	CheckMate-9LA, [299]
NSCLC-squamous	Metastatic, first-line	Carboplatin + paclitaxel/ nabpaclitaxel	Pembrolizumab	OS: 15.9 versus 11.3 months	HR 0.64; 95% CI 0.49 to 0.85; *P* < 0.001	KEYNOTE-407, [52]
SCLC	Extensive stage, first-line	Carboplatin + etoposide	Atezolizumab concurrent and maintenance	OS: 12.3 versus 10.3 m	HR 0.70; 95% CI 0.54–0.91;* P* = 0.007	IMpower133, [53]
SCLC	Extensive stage, first-line	Carboplatin + etoposide	Durvalumab	OS: 12.9 versus 10.5 months	HR 0·73 (95% CI 0·59–0·91; *p* = 0·0047	CASPIAN, [54]
Breast triple negative	Metastatic, first-line	nabpaclitaxel	Atezolizumab	OS: 25.0 versus 15.5 months (PD-L1( +)	HR 0.62; 95% CI 0.45–0.86	IMpassion 130, [55]
Breast triple negative	Metastatic, first-line	Nabpaclitaxel or paclitaxel or carbpolatin + Gemcitabine	Pembrolizumab	PFS (CPS > 10) 9.7 versus 5.6 m:	HR 0·65, 95% CI 0·49–0·86; one-sided *p* = 0·0012	KEYNOTE 355, [56]
Bladder cancer	Metastatic, first-line maintenance	Gemcitabine + cisplatin/carboplatin	Avelumab	OS: 21.1 versus 14.3 Mo	HR 0.69; 95% CI 0.56 to 0.86; *P* = 0.001	JAVELIN Bladder 100, [58]
Head and Neck Cancer	Metastatic first-line	Platinum + 5-FU or platinum + 5-FU + cetuximab	Pembrolizumab	OS: 13·6 versus 10·4 (CPS ≥ 1)	HR 0·65; 95% CI 0·53–0·80; *p* < 0·0001	KEYNOTE-048, [57]

In small cell lung cancer (SCLC), two immune checkpoint inhibitors, atezolizumab and durvalumab, were approved with the combination of standard of care platinum-based chemotherapy [53, 54]. The addition of atezolizumab improved the OS from 10.3 months to 12.3 months (HR 0.70, *P* = 0.007), while the addition of durvalumab improved the OS from 10.3 months to 13.0 months (HR 0.73, *P* = 0.0047).

In addition to lung cancers, the combination of chemotherapy and immunotherapy has also been approved in several other cancers. In breast cancer, atezolizumab plus nab-paclitaxel improved the OS of the intended population from 17.6 months of nab-paclitaxel alone to 21.3 months (HR 0.84; 95% CI, 0.69–1.02; *p* = 0.08) [55]. Furthermore, the addition of pembrolizumab to chemotherapy improved median progression free survival (PFS) from 5.6 months to 9.7 months in the population with PD-L1 expression at a combined positive score of 10 or higher (HR 0·65, 95% CI 0·49–0·86; one-sided *p* = 0·0012) [56]. In head and neck cancer, the addition of pembrolizumab to cisplatin/carboplatin + 5-fluouracil significantly improved OS when compared to the addition of cetuximab to chemotherapy in the group with the PD-L1 combined positive score of 1 or higher: median OS 13·6 versus 10·4 months (HR 0·65, 95% CI 0·53–0·80, *p* < 0·0001) [57].

The OS benefit has also been observed when immunotherapy was used as a maintenance therapy after completion of chemotherapy, as in bladder cancer. In the JAVELIN Bladder 100 trial, significantly improved OS was observed in patients with metastatic urothelial carcinoma who completed platinum-based chemotherapy without disease progression was subsequently treated with avelumab maintenance therapy: median OS 21.4 versus 14.3 months (HR 0.69, 95% CI 0.56–0.86, *p* = 0.001) [58].

However, chemo-immunotherapy combinations have not been a panacea in all solid tumors. In squamous NSCLC, even though the combination of pembrolizumab and chemotherapy improves OS, the addition of atezolizumab to chemotherapy did not (14.2 and 13.5 months, HR 0.88, 95% CI 0.73–1.05, *p* = 0.16) [59]. In metastatic urothelial cancer, chemo-immunotherapy combinations have been disappointing with minimal improvements over chemotherapy alone, in contrast to the Javelin Bladder 100 trial, where avelumab maintenance therapy significantly improved treatment outcomes. In part, this is likely due to patient selection from patients initially doing well after chemotherapy selected for the Javelin Bladder 100 trial and not delaying treatment until progression. More studies are needed to determine the optimal combination, sequence, drug choice and underlying mechanisms of different response.

## Combination of radiation therapy with immunotherapy

The stimulation of anti-cancer immunity by radiotherapy (RT) was first suggested in case reports with regression of distant untreated tumors after local RT [60]. While this RT-induced abscopal phenomenon is rare and elusive, its effects on the induction of anti-cancer immune response are intriguing and have aroused tremendous interest with the advent of immune checkpoint blockade.

### Potentiation of anti-cancer immunity by radiation

Both antigenicity and adjuvanticity are critical for immune response. RT can augment both antigenicity and adjuvanticity in addition to alteration of the local TME.

RT increases tumor antigenicity through multiple pathways. First, similar to chemotherapy as discussed above, radiation can induce MHC-I expression and enhance tumor antigen presentation [61]. Second, radiation induces ICD. During ICD, annexin A1 guides antigen-presenting cells to dying cancer cells while HSP70, HSP90, HMGB1 and other molecules promote uptake and cancer antigen presentation to T cells. It has been shown that radiation induces translocation of calreticulin to the plasma membrane [62], and release of HMGB1 [63]. Third, radiation downregulates CD47 expression on the cell surface and enhances the cancer cells’ uptake and antigen presentation [64]. CD47 presents as a “do not eat me” signal to APCs and is overexpressed in many cancer cells [65]. Fourth, reactive oxygen species (ROS) generated during ionizing radiation can modify macromolecules, such as proteins and DNA, and increase antigenicity. In addition to direct DNA damage, the presence of oxygen and generation of ROS are critical for radiation induced tissue injury [66].

Another important contribution of radiation to anti-cancer immunity is increased adjuvanticity. Radiation-induced DNA damage and cytoplasmic leakage of DNA from micronuclei activate the innate and adaptive immune response via cGAS/STING pathway and upregulate the expression of type I interferon pathway. This pathway is critical for radiation induced anti-cancer immunity. Silencing of cGAS in bone marrow-derived dendritic cells impairs T cell priming [67]. In addition to nuclear DNA, mitochondrial DNA breaks also have a role in activating a type I interferon response and synergizing with nuclear DNA breaks [68].

In addition to the cGAS-STING pathway, ICD, release of DAMPs and cytokines can enhance adjuvanticity, elicit migration of pro-anti-cancer immune subpopulation, decrease immunosuppressive cells, alter TME and tilt immune response to cancer cell killing. Overall, radiation converts cancer cells as an in situ vaccine to elicit anti-cancer immunity.

### Inhibition of anti-cancer immunity by radiation

In contrast to what is discussed above, ample evidence also exists that radiation induces an immunosuppressive TME. In addition to cancer cells, radiation can kill normal cells, including immune cells, especially when broad field radiation is considered. Furthermore, radiation can alter the TME and, instead of tilting to anti-cancer immunity, induce an immunosuppressive milieu. Several studies showed that radiation induces infiltration and aggregation of MDSCs [69, 70], which contributes to the immunosuppressive TME through multiple pathways. The same STING pathway that contributes to the cancer adjuvanticity at least partially contributes to the aggregation of MDSCs in tumor tissues [71]. In addition, radiation can promote the expression of TGF-β and TGF-β family activin A, thus promoting the recruitment of T_reg_ cells and reducing the infiltration of CD8^+^T cells [72]. TGF-β is upregulated upon radiation [73]. In a preclinical study, TGF-β neutralization and radiation increase T cell priming and decrease tumor growth and metastasis [74].

Other mechanisms of the immunosuppressive effects of radiation include the dysregulation of tumor blood vessels [75], hypoxia [76], stroma [77], tumor-associated macrophages (TAMs) [78], cancer-associated fibroblasts (CAFs) [79], cytokines [80, 81] and so on. Moreover, the abnormal expression of these components is also related to radiation resistance [82]. In conclusion, the formation of an immunosuppressive TME by radiation is a complicated process and targeting these immunosuppressive elements provides a new direction for enhancing RT-induced anti-tumor immunity.

### Clinical consideration of radiation and immunotherapy combination

The first report showing the benefits of radiation and immunotherapy came from a patient with melanoma who had disease progression while on a clinical trial with ipilimumab, but subsequently had abscopal tumor shrinkage after radiation therapy [83]. A secondary analysis of the KEYNOTE-001 trial also showed that prior radiotherapy is associated with significant improvement of PFS and OS of patients with NSCLC treated with pembrolizumab [84]. Since then, there has been an eruption of clinical trials with radiotherapy and immunotherapy. Currently, over 800 active clinical trials are registered at clinicaltrials.gov, when using radiation and immunotherapy as the search key words.

So far, several clinical studies showed improved clinical outcomes when radiation is added to ICBs. In a meta-analysis including 20 clinical trials and 2,027 NSCLC patients, the combination of anti-PD1/PD-L1 inhibitors with radiotherapy was associated with significantly improved objective response rate (odds ratio [OR] 2.76, 95% CI 1.06–7.19, *p* = 0.038) and OS (2-year survival HR 1.77, 95% CI 1.35–2.33, *p* = 0.000) [85]. Currently, durvalumab has been approved as a maintenance therapy after platinum-based chemoradiation therapy for stage III NSCLC patients based on a Phase III PACIFIC trial [86, 87]. Addition of durvalumab significantly increased the median PFS (17.2 vs. 5.6 months; HR 0.51, 95% CI 0.41–0.63, *p* < 0.001) and OS (HR for death 0.68, 95% CI 0.54–0.86, *p* = 0.0025).

In addition to anti-PD1/PD-L1 antibodies, radiotherapy is already being studied with the combination of other immunotherapeutic agents such as cytokines, cell therapy, vaccines and other immune checkpoint modulators [88]. While most of these studies are still ongoing, some early reports show that these combinations are feasible and can potentially achieve synergistic effects. In a small Phase II trial, radiotherapy combined with CAR-T cell therapy improved the overall RR of diffuse large B-cell lymphoma (*p* = 0.033) [89].

Even though promising results were observed, other studies showed no improvement with the radioimmunotherapy combination. Several approaches are currently being explored to improve treatment outcomes. Selection of the right patients (biomarker development) and optimization of radiation techniques, including dose, schedule and timing, are both under intense investigation. Preclinical and clinical data suggest that dose and fraction, irradiated area, volume and sequence of administration can each have major impact in systemic anti-cancer immunity [90, 91]. Because radiation not only kills cancer cells, but also affects many aspects of immune response, such as cancer antigenicity, presentation, TIME, immune response at local drainage lymph nodes and in the whole system, it is not surprising that contradictory findings were observed regarding anticancer immunity with different dose and fractionation schedules. Lymphocytes have little DNA repair capacity and are highly sensitive to radiation even at the conventional dose of 1.8–2 Gy [92]. One study showed post-radiation immune cell re-population differs with lymphoid response observed more with hypofractionation while conventional dose/schedule induces more myeloid response, such as MDSCs and TAMs [93]. Several preclinical studies revealed that high-dose hypofractionation radiation stimulates more anticancer immune response than conventional fractionation radiation. High-dose radiation increases expression of MHC and death receptors critical for T cell-mediated cell killing [61], induces more T cell infiltration into tumors [94], triggers more robust abscopal effects [95] and synergizes more with anti-PD-L1 and anti-TIGIT therapies [93]. A US national database analysis also revealed that hypofractionated radiation therapy and immunotherapy achieved much higher three-year overall survival in metastatic melanoma patients than conventionally fractionated radiation plus immunotherapy (37.3% vs. 17.6%, *p* < 0.0001) [96]. However, less favorable results with high-dose hypofractionation were also observed in other preclinical studies. In a breast cancer model, the abscopal effect was only observed when anti-CTLA-4 therapy was combined with fractionated radiotherapy, but not with single high-dose therapy [91]. High-dose radiation induces DNA exonuclease Trex1 and dampens the cGAS-STING pathway activation [97]. Hence, well-designed prospective clinical trials are needed to determine the optimal radiation dose, schedule and fractionation to potentiate immunotherapy.

## Combination of targeted therapy with immunotherapy

All cancers harbor genomic alterations that drive oncogenesis. Targeting these genomic alterations can have direct antitumor activities and can induce more responses than cytotoxic chemotherapy [98, 99]. For example, in patients with NSCLC, while the response rate of platinum-based doublet is less than 30% [100], a response rate of 80% is observed in patients with an epidermal growth factor receptor (EGFR) driver mutation treated with erlotinib [101]. In addition, many of the molecular drivers affect multiple steps along the cancer-immunity cycle.

### Potential mechanisms

#### Direct antitumor activity and ICD

Elimination of cancer cells can not only decrease the number of cells for immune cells to target and destroy, but can also eliminate immunosuppressive factors and increase the efficacy of immunotherapy. The KEYNOTE-001 trial showed that smaller tumor sizes are an independent factor in predicting treatment outcomes [102]. An important factor to consider is ICD induced by targeted therapy. As discussed above in the sections of chemotherapy and radiation therapy, ICD induced by targeted therapy enhances cancer cell uptake and antigen presentation by antigen-presenting cells, prime and activate immune response, attract immune cells to tumor sites and potentiate anti-cancer immunity.

#### Antigen presentation

Many of the oncogenic pathways are directly involved in the regulation of the expression of antigen presentation machinery. The cyclin-dependent kinase 4 and 6 (CDK4/6) pathway is commonly activated in many cancers [103, 104]. Inhibition of the CDK4/6 pathway upregulates MHC expression [103]. Similar findings are also observed with the PI3K pathway. PI3K inhibitors have been approved in breast cancer and follicular lymphoma. These drugs have the potential to be effective in other cancers, such as bladder cancer [98, 99]. Activation of the PI3K pathway attenuates the expression of MHC class I and II expression, while inhibition of this pathway reverses the suppression of antigen presentation machinery via interferon γ [105].

#### Direct effect on immune cells

Many of the aberrant signaling activities have profound impacts on immune cells. The VEGF-VEGFR pathway plays critical roles in almost every subpopulation of immune cells. VEGFRs are expressed on activated and memory T cells [106]. Engagement of VEGF-VEGFR leads to activation of the downstream signaling pathways in T cells [106], inhibits TCR (T cell receptor)-dependent activation in T cells [107] and suppresses the cytotoxic activity of T cells [108]. In T_reg_ cells, VEGFR2 is selectively expressed in FOXP3^high^ T_reg_ cells. Besides T_reg_ cells, VEGF can activate JAK2 and STAT3 and induce accumulation of Gr1 + CD11b + MDSCs [109]. In dendritic cells, production of VEGF by human tumors inhibits dendritic cell maturation through the NF-kappa B pathway [110, 111]. Increased plasma VEGF levels are associated with increased number of immature dendritic cells, and surgical removal of tumors partially reverses these effects [112]. In applying these preclinical findings to clinical trials, the combination of angiogenesis inhibitors and ICB significantly improved the treatment outcomes in metastatic renal cell carcinoma and has gained several FDA approvals.

Similar direct effects on immune cells are also seen with many other targeted agents already approved by the FDA or in development. For example, ibrutinib is FDA approved for chronic lymphocytic leukemia/lymphoma (CLL), mantle cell lymphoma, marginal zone lymphoma and Waldenström’s macroglobulinemia. It modulates T cells by inhibiting Bruton's tyrosine kinase (BTK) and IL-2-inducible T cell kinase (ITK), and drives a Th1-selective pressure in T lymphocytes and a preferential inhibition of Th2 response [113]. In patients with CLL, it markedly increases CD4 + and CD8 + T cell numbers, decreases T_reg_/CD4 + T cell ratio, downregulates immunosuppressive CD200 and CD272 expression and decreases the production of immunosuppressive IL-10 production. Currently, seven clinical trials are ongoing to combine ibrutinib with immune checkpoint inhibitors for treatment of cancer.

#### Effects on tumor microenvironment

In addition to the direct antitumor activity, many of the signaling pathways have versatile functions on the tumor immune microenvironment (TIME) that can affect anti-cancer immunity. EGFR activation mutations occur in about 10–15% of all NSCLC and can upregulate PD-1 and PD-L1 expression which mediates immune escape [114]. Similarly, activation of the PI3K/AKT pathway, including the *PTEN* deletion, leads to constitutive expression of PD-L1 expression and resistance to immunotherapy [115].

Other than immune cells, no other cells play such a versatile array of functions in TIME as CAF [116, 117]. CAFs can secrete immunosuppressive cytokines, attract suppressive immune cell subpopulations, remodel tumor matrix and facilitate migration, invasion and metastasis of cancer cells. CAFs can alter local milieu and indirectly suppress anti-cancer immunity, and facilitate tumor cell growth [118]. Cross-communication between cancer cells and CAFs contributes to development of resistance to chemotherapy and immunotherapy. Many of the altered signaling transduction pathways, such as receptor tyrosine kinase receptors and their cognate ligands, Wnt signaling pathway, TGF-β pathway and others, contribute to activation of fibroblasts to CAFs [118, 119].

Many of the genomic alterations and oncogenic drivers alter metabolism and other constitutive components of the tumor microenvironment and negatively affect anti-cancer immunity. Oncogenic transformation leads to uncontrollable cancer cell proliferation, creates hypoxic and acidic tumor microenvironments and inhibits T cell function. Indolamine-2,3-dioxygenase (IDO) is a heme-containing enzyme that catalyzes the first and rate-limiting step of tryptophan catabolism. Depletion of the essential amino acid tryptophan and accumulation of the metabolic products, such as kynurenine, are highly immunosuppressive and tolerogenic. They can suppress effector T cell and NK cell function, stimulate T_reg_ cells, promote expansion of MDSCs and tilt polarization of macrophages to more tolerogenic M2 phenotype [120]. It has been shown that multiple oncogenic pathways, such as the PI3K/AKT/mTOR, Ras/Raf/MEK/ERK and protein kinase C pathways, are all involved in the upregulation of IDO expression [121].

### Clinical consideration of the targeted therapy and immunotherapy combination

Since many of the targeted therapeutic agents can directly or indirectly modulate immune cell functions, a plethora of clinical trials are currently ongoing to determine the efficacy and toxicity of combined targeted therapy and immunotherapy, mainly ICBs, in cancer [122–124]. As discussed above, anti-angiogenesis agents probably have the most versatile immune-modulative functions that affect almost all immune cell subpopulations [125]. Hence, it is not surprising that five out of six FDA-approved targeted and immunotherapy combinations target angiogenesis (Table 4): axitinib targets VEGFR 1–3 in addition to platelet-derived growth factor receptor (PDGFR) and c-Kit; cabozantinib inhibits VEGFR2 in addition to c-Met and Axl; lenvatinib targets VEGFR1-3 in addition to fibroblast growth factor receptors, PDGFR and RET; and bevacizumab is a monoclonal antibody against VEGF-A. The only targeted therapy combination that does not directly target angiogenesis is the combination of a BRAF inhibitor vemurafenib and a mitogen-activated extracellular kinase (MEK) inhibitor cobinetinib in combination with atezolizumab in advanced melanoma with BRAF V600 activation mutation. Three of the six combinations are indicated for advanced kidney cancer: pembrolizumab plus axitinib, avelumab plus axitinib and nivolumab plus cabozantinib.

**Table 4:** FDA-approved combination regimens of immunotherapy and targeted therapies

Cancer	Line of therapy	Targeted therapy	Immunotherapy	Clinical benefit	Statistics	Trial name and reference
Kidney cancer	Metastatic, 1st line	Axitinib	Pembrolizumab	12-Mo OS: 89.9% versus 78.3%	HR 0.53; 95% CI 0.38 to 0.74; *P* < 0.0001	KEYNOTE-426, [301]
Kidney cancer	Metastatic, 1st line	Cabozantinib	Nivolumab	PFS 16.6 versus 8.3	HR 0.51; 95% CI 0.41 to 0.64; *P* < 0.001	CheckMate -9ER, [303]
Kidney cancer	Metastatic, 1st line	Axitinib	Avelumab	PFS 13.8 versus 7.2 mos,	HR 0.61; 95% CI, 0.47 to 0.79; *P* < 0.001	JAVELIN Renal 101, [302]
Endometrial cancer not MSI-H or dMMR	Metastatic, salvage	Lenvatinib	Pembrolizumab	ORR of 38.3% (95% CI, 29–49%)	Single-arm trial	KEYNOTE-146, [306]
Hepatocellular carcinoma	Unresectable, 1st line	Bevacizumab	Atezolizumab	12-mo OS: 67.2% versus 54.6% for sorafenib	HR 0.58; 95% CI 0.42 to 0.79; *P* < 0.001	IMbrave150, [309]
BRAF V600( +) advanced melanoma	Advanced, 1st line	Vemurafenib + cobimetinib	Atezolizumab	PFS 15.1 versus 10.6 mo	HR 0·78; 95% CI 0·63–0·97; *p* = 0·025	IMspire150, [313]

In addition to anti-angiogenesis, almost every targeted therapy that has been shown to modulate the immune response is currently being combined and tested with immunotherapy, mainly ICBs. For example, PI3K inhibitors have been approved for the treatment of breast cancer and lymphoma. In addition to direct anti-cancer activity, it alters tumor local metabolism, downregulates antigen presentation machinery and has direct effects on immune cells as PI3K-δ is expressed in immune cells [126]. Over 10 clinical trials are currently ongoing that combine immunotherapy with agents targeting the PI3K/AKT/mTOR pathway [127].

In addition to ICBs, targeted therapy is also being combined with other immunotherapeutic agents. The BTK/ITK inhibitor, ibrutinib and acalabrutinib have been approved for the treatment of non-Hodgkin lymphoma and are known to increase T cell number and function. Currently, seven clinical trials have been designed to combine the BTK/ITK inhibitors with CAR-T cell therapy and one clinical trial combining ibrutinib with personalized multi-peptide cancer vaccine.

## Cytokines and other soluble factors

Cytokines are small proteins or glycoproteins (< 30 KDa) that interact with cell surface receptors and exert critical roles in regulating humoral and cellular immune response through affecting cell trafficking, maturation, growth and responsiveness of target cells. Cytokines include chemokines, interleukins, interferons and tumor necrosis factors. Interleukin-2 (IL-2) and interferon α (IFN-α) are the first two cytokines approved for the treatment of cancers.

### Chemokines

Chemokines are the largest subfamily of cytokines that play important roles in guiding immune cell trafficking and development, and can be classified into four main classes depending on the location of the first two cysteine (C) residues in their protein sequence: namely, the CC-chemokines, the CXC-chemokines, C-chemokines and CX3C-chemokines [128].

Different immune cell subpopulations respond to different chemokines, traffic into TME and affect anti-cancer immunity. For example, effector immune cells, such as CD8 + T_eff_ cells, IFN-γ-expressing T helper 1 (TH1) cells and natural killer (NK) cells, can be attracted to the tumor microenvironment by CXC-chemokine ligand 9 (CXCL9), CXCL10 and CXCL11, and exert potent antitumor effects [129, 130]. T_reg_ cells are immunosuppressive cells that can inhibit the functions of other immune cells through interaction of inhibitory cell surface receptor, such as: CTLA4-CD28 interaction [131], CTLA4-CD80/86 and LAG3/MHCII pairs; secretion of inhibitory cytokines such as TGF-β, IL-10 and IL-35; secretion of granzyme B and lysis of T_eff_ cells; and metabolic disruption such as the adenosine pathway [132]. T_reg_ cells express CCR4 and CCR10, and migrate into the tumor microenvironment in response to CCL22 and CCL28 [133, 134]. Dendritic cells can be attracted by CCL5, CCL20 and CXCL12 [135], while macrophages can be attracted by the CCL2-CCR2 signaling [136]. Sometimes the same chemokines can recruit different immune cells with different and even opposing immune functions. For example, CCL21 and CCL19 recruit CCR7 + dendritic cells and T_reg_ cells, while CCL17 and CCL22 can directly recruit T_reg_ and Th2 lymphocytes [137–140].

In addition to regulating immune cell trafficking and development, chemokines have direct effects on cancer cells. Cancer cells can express chemokine receptors and be stimulated by chemokines to promote cancer cell growth and proliferation [141–143]. Furthermore, chemokines can also facilitate cancer metastasis. The CXCL12/CXCR4 and CCL27/CCR10 pathways have both been found to be involved in cancer cell adhesion, migration and metastasis [144–146].

### Interleukins and interferons

While the major function of chemokines is to regulate immune cell trafficking, interleukins and interferons have diverse functions in regulating the immune response. IL-2 and IFN-α were the first cytokines approved for cancer therapy. Since the first approval for the treatment of hairy cell leukemia [147], IFN-α has also been approved for the treatment of melanoma, follicular non-Hodgkin’s lymphoma, AIDS-related Kaposi’s sarcoma and renal cell carcinoma, while IL-2 was approved for the treatment of renal cell carcinoma and melanoma[148].

Pro-inflammatory interleukins and IFN-α act upon every step of the cancer immunity cycle and promote anti-cancer immunity, while immunosuppressive cytokines promote many aspects of oncogenesis and inhibit anti-cancer immunity. IL-2 plays key roles in the expansion of T lymphocytes and NK cells. T_reg_ cells have high expression of IL-2 receptor alpha (IL-2Rα), which is a component of high-affinity IL-2 receptor. Hence, IL-2 can skew the expansion of T lymphocytes to T_reg_ cells [149, 150]. To generate more favorable immune cell stimulation and decrease T_reg_ cell proliferation, several strategies have been used to modify IL-2. One strategy is to conjugate recombinant IL-2 with polyethylene glycol (PEG), as in NKTR-214 (or bempegaldesleukin), that decreases the affinity to the high-affinity IL-2Rα receptor. Another strategy is to introduce a mutation to IL-2 to decrease its binding to IL-2Rα, known also as CD25. Both versions are currently in clinical development [151, 152].

### Transforming Growth Factor-β (TGF-β)

TGF-β is a pleiotropic cytokine that plays key roles in embryogenesis and tissue homeostasis. It regulates cell proliferation, differentiation, adhesion, migration, metabolism and apoptosis in many normal cells. At early stage of oncogenesis, TGF-β can inhibit cancer growth, induce apoptosis and work more like a tumor suppressor. Once cancer develops, it is involved in promoting tumor fibrosis, epithelial–mesenchymal transition (EMT), tumor angiogenesis and suppression of immune response [153, 154]. Tumor fibrosis can prevent drugs and immune cells from accessing cancer cells, while EMT can lead to metastasis and resistance to therapy. Furthermore, TGF-β regulates many immune cell subtypes and is intensively involved in the immunosuppressive TME. It can suppress the expression of IL-2 which is critical for T cell proliferation [155], inhibit the differentiation of naïve T cells into Th1 cells [156] and mitigate the cytotoxic effects of CD8 + T_eff_ cells through inhibiting the expression of five cytolytic gene products—namely, perforin, granzyme A, granzyme B, Fas ligand and interferon γ [157]. For T_reg_ cells, TGF-β triggers the expression of FOXP3 which serves as the master transcription regulator for the T_reg_ cell differentiation. Furthermore, T_reg_ cells can carry latent TGF-β1, as well as a cell surface docking receptor GARP for latent TGF-β, to suppress anti-cancer immunity [158–163].

In addition to T cells, TGF-β has immunosuppressive effects on many other immune cells. In dendritic cells, TGF-β suppresses expression of MHC-II genes and inhibits antigen presentation [164, 165]. For natural killer cells, TGF-β blocks NK functions by decreasing the expression of NK cell surface receptors NKG2D and NKp30 [166], and inhibits Th1 response through suppressing IFN-γ and TBET expression [167, 168]. For macrophages, TGF-β induces macrophage differentiation to the M2 phenotype that is immunosuppressive in the TME [153, 169]. Furthermore, TGF-β plays important roles in tumor development and metastasis via MDSCs. Depletion of MDSCs abolishes the therapeutic effects of an anti-TGF-β antibody, at least in preclinical studies [170].

In addition to direct effects on immune cells, TGF-β plays major roles in the immunosuppressive TME. TGF-β produced by CAFs excludes CD4 + and CD8 + T cells from entering the tumor [171] and an anti-TGF-β antibody could reverse tumor T cell exclusion and sensitize tumors to PD-L1 treatment [172]. TGF-β produced in the TME can induce the expression of indoleamine 2,3-dioxygenase (IDO) and arginase which can suppress many effector immune cells [173].

Because of the pluripotent regulation of anti-cancer immunity functions by TGF-β, multiple therapeutic agents targeting TGF-β have been developed and are currently in clinical development (Table 5). Given the importance of TGF-β and its effects in the TME, Dr. James Gulley at the National Cancer Institute discussed the pathway and highlighted bintrafusp alfa at the 2020 China IO Meeting. Bintrafusp alfa is a bifunctional chimeric protein composed of the extracellular domain of the TGF-β receptor II (a TGF-β “trap”) fused to anti-PD-L1 human IgG. It is hypothesized that bintrafusp alfa carries the TGF-β trap to the cancer sites where PD-L1 is expressed, blocks both TGF-β and PD-L1, and enhances anti-cancer immunity. Preclinical studies showed significant anti-tumor effect, TME modification and reduction of EMT [174, 175]. A Phase I trial with bintrafusp alfa was conducted which showed promising anti-tumor effects with controllable toxicities [176]. So far, 30 clinical trials have been registered at clinicaltrials.gov with bintrafusp alfa in various cancers.

**Table 5:** Therapeutic strategies targeting the TGF-β pathway

Therapeutic categories	Targets	Drugs
Small molecules	TGF-βR1	Galunisertib, vactosertib, BMS-986260, LY3200882; PF-06952229
Antibodies	Pan-TGF-β	Fresolimumab, SAR439459, NIS793
	Glycoprotein-A repetitions predominant (GARP)- TGF-β1	ABBV-151
	TGF-β1 and TGF-β2	XPA-42-089
Bi-specific antibodies	TGF-βRII and PD-L1	Binstrafusp alfa
	TGF-βRII and CTLA4	*a*-CTLA4-TGFβRII*ecd*
Antisense	TGF-β2	Trabedersen
Modified ACT	Dominant-negative TGF-βRII	

### Strategies for clinical use and combination therapy

At the 2020 China IO meeting, Dr. Charles Drake from Columbia University and Dr. James Gulley from the National Institute of Health discussed strategies to target cytokines and other combinations for cancer immunotherapy. Dr. Drake first discussed the serendipitous findings from a clinical trial with an anti-IL-1β monoclonal antibody, canakinumab, in preventing cardiovascular diseases. People treated with canakinumab at 300 mg every 3 months had a relative risk of overall cancer incidence of 0.49 and fatal lung cancer of 0.23 when compared to the placebo cohort [177], suggesting that canakinumab has a protective effect. His group then confirmed that an anti-IL-1β antibody, especially in combination with anti-PD1 antibody, dramatically increased M1 macrophage and the M1/M2 macrophage ratio in the TME [178]. Subsequently, a pilot clinical trial was initiated to determine the efficacy and molecular correlative studies in kidney cancer (ClinicalTrials.gov Identifier: NCT04028245). He also discussed that cytokines can be significantly affected by androgen deprivation therapy (ADT) in prostate cancer that can possibly be targeted for cancer therapy. In mice, ADT significantly increases the expression of CXCL15, which is the mouse equivalent of human IL-8. This cytokine pathway is involved in infiltration of neutrophils and polymorphonuclear myeloid-derived suppressor cells (PMN-MDSC) into the immunosuppressive TME. Based on those findings, a clinical trial was initiated with the anti-PD1 antibody nivolumab in combination with an anti-IL-8 antibody to synergize ADT in prostate cancer (Clinicaltrials.gov identifier No: NCCT03689699).

As discussed above, IL-2 and IFN-α are rarely used in clinic due to their systemic pro-inflammatory side effects. One future development strategy to use cytokines for cancer immunotherapy is to confine cytokines to the site of action, such as intratumoral injection of the cytokines or using gene therapy or other vehicles to express cytokines into the cancer sites. Intratumoral injection of IL-2 and IFN is one of the earliest formats of targeted delivery of cytokines to the site of action to minimize systemic pro-inflammatory reaction. Talimogene laherparepvec (TVEC) is a genetically engineered oncolytic herpes virus expressing human granulocyte–macrophage colony-stimulating factor (GM-CSF) that has been approved for intratumoral injection of melanoma [179].

Since intratumoral injection may not be practical in patients with multiple metastatic lesions or deep locations of cancer, another strategy is to modify cytokines and change their binding specificity. NKTR-214, a therapeutic where IL-2 is conjugated to polyethylene glycol (PEG), has decreased affinity for the high-affinity IL-2 receptor α and therefore lower associated toxicities compared to IL-2 [180]. Consistent with the findings in preclinical models, NKTR-214 significantly promotes cytotoxic immune cell infiltration and upregulates gene expression associated with effective cells with limited increase of T_reg_ cells in tumors in a Phase I clinical trial [181], with further clinical development in urothelial and renal cancers.

More recently, cytokine-based bifunctional molecules have generated great interest in which the cytokine in the molecule exerts its immunoregulatory functions while the other part of the molecule acts as a carrier to deliver the cytokine to the site of action as seen in RO6874281, or as a carrier and functional domain as seen in bintrafusp alfa discussed above. RO6874281 contains a variant form of interleukin-2 (IL-2v) that completely lacks binding to the high-affinity IL-2 receptor α, but retains IL-2Rβγ binding. IL-2v is conjugated to a human monoclonal antibody directed against fibroblast activation protein-alpha (FAP) on CAF [182]. Both RO6874281 and bintrafusp alfa have shown clinical activities in addition to their reduced toxicity [182, 183]. In a Phase I trial with bintrafusp alfa as a second-line treatment for NSCLC, an overall response rate of 21.3% (17 of 80) was observed in the whole study population. It was 25.0% (10 of 40) at the recommended Phase 2 dose of 1200 mg every 2 weeks and 36.0% (10 of 27) in those with PD-L1-positive tumors [183].

Because cytokines can regulate every step along the anti-cancer immunity cycle, many clinical trials are ongoing to combine cytokines with other agents along the immunity cycle to determine whether the anti-cancer efficacy can be further improved. For immunostimulatory cytokines, such as IL-2, IL-10, IL-12 and IL-15, their native forms and genetically engineered cytokines have been combined with ICBs. For example, the combination of pegylated long-acting IL-10 and anti-PD1 antibody pembrolizumab or nivolumab had manageable toxicity profiles and showed preliminary antitumor activity [184]. For immunosuppressive cytokines, such as TGF-β, CCL2 and IL-8, their neutralizing antibodies or small molecule inhibitors have been tested in clinic with the combination of ICBs and chemotherapy.

## Adoptive cell therapy

### Brief history

Adoptive cell therapy (ACT) in cancer is the transfer of immune cells, either autologous or allogeneic, into patients with cancer to mount an anti-cancer immune response. In both cases, immune cells are isolated from patients themselves (autologous) or a donor (allogeneic), manipulated and expanded in vitro, and infused into patients for cancer therapy. The first ACT was performed in patients with metastatic melanoma using autologous tumor-infiltrating lymphocytes (TILs) [185]. TILs are available in only a minority of patients with selected tumors, usually melanoma, and associated with inconsistent response rates. With the development of chimeric antigen receptor (CAR) technology [186–188], melanoma tumors were shown to clinically regress after infusion of normal lymphocytes expressing an engineered T cell receptor targeting the MART-1 tumor antigen [189]. The research and clinical applications accelerated after 2010 with the demonstration of tumor regression in B cell lymphoma after administration of lymphocytes expressing CAR against the B cell antigen CD19 [190].

Because NK cells mirror the functions of CD8 + cytotoxic T cells [191], NK cells have also been engineered to express CARs for cancer immunotherapy [192]. The cytotoxic function of NK cells is upregulated via engagement of activating receptors, such as NKG2D. Hence, CAR NK cells usually use one of these activating receptors such as CAR NK cells expressing NKG2D-containing CARs [193, 194]. So far, several clinical trials with CAR NK cells targeting hematological malignancies (CD7, CD19, CD22, CD33, BCMA) and solid tumors (Robo1 and MUC1) are ongoing (www.clinicaltrials.gov).

### Resistant mechanisms

Even with great success and FDA approvals of ACT, especially in hematological malignancies, 10–20% patients fail to achieve remission after receiving anti-CD19 CAR-T cell therapy, and 30–50% who achieve initial remission develop disease relapse [195, 196]. Some of the treatment failure can be secondary to logistic issues, such as manufacturing failure and delay, insufficient numbers of CAR-T cells, delay in insurance approval and disease progression to an irreversible end stage. More commonly, the same mechanisms of resistance to anti-cancer immunity, especially those at TME, are responsible for resistance to ACT.

CAR-T cells bypass the first three steps of the cancer immunity cycle: antigen release and presentation, immune cell priming and immune cell activation. However, like any other effector immune cells, ACT is still governed by the regulation of immune response and resistant mechanisms along the anti-cancer immunity cycle described above [3]. Since CAR-T cells are engineered T cells, the intrinsic T cell function status can affect the treatment outcomes. After infusion, CAR-T cells have 3 main characteristics to achieve long-lasting remission: expansion, persistence and tumor cytotoxicity. Hence, defects of the original T cells that affect T cell expansion, cytotoxic function and development of memory cells can also affect the efficacy. For example, the efficacy of ACT is inferior in chronic lymphocytic leukemia (CLL) than that in B-cell acute lymphoblastic leukemia (B-ALL), which may be related to the intrinsic T cell defects in CLL patients. Hence, generation of universal CAR-T cells from healthy donors or third-party donors is being explored [197, 198].

CAR-T cells contain a T cell receptor stimulatory domain and a co-stimulatory domain, both of which are required for T cell priming, activation and replication. Preclinical and observation studies showed that the co-stimulatory domain can significantly affect the persistence and cell function after infusion [199]. Optimization of CAR design to enhance CAR-T cell activation, replication and conversion to memory cells is ongoing. Compared to the second-generation CAR which contains a single costimulatory domain (CD28, 4-1BB or OX-40), the third-generation CAR contains two or more costimulatory domains which can exhibit strong short-term anti-tumor activity associated with CD28 and long-term persistence with 4-1BB [200, 201].

After infusion, CAR-T cells still need to go through cell trafficking, infiltration into the cancer sites and then recognition and killing of cancer cells. Dysregulation of cytokine and cytokine receptors, and an immunosuppressive TME can adversely affect CAR-T cell function. It has been shown that β-catenin- over-expressing tumors have an altered CXCR3-CXCL9/10 chemokine axis to attract effector T cells into tumors after adoptive transfer [202]. Delivery of CXCL11 to tumor sites significantly increases CAR-T cell infiltration and enhances anti-tumor activity [203].

Once inside tumors, suppressive signals produced in the TME, such as TGF-β, IDO1, IL-10 and adenosine, contribute to exhaustion of CAR-T cells. Tumor cells can produce suppressive signals, such as PD-L1, that suppress CAR-T cells. CAFs, MDSCs and TAMs can all contribute to the suppression of CAR cell function in the TME. Combination of ACT with other therapies to prevent exhaustion and enhance CAR-T cell functions is being explored. Blockade of adenosine 2A receptor significantly improves the efficacy of CAR-T cells [204]. PD1 is another negative regulator of T_eff_ cells at the end cytotoxic stage and was found to be upregulated after CAR-T cell infusion [205].

In addition to the mechanisms along the cancer immunity cycle, alterations in malignant cells also contribute to primary and secondary resistance to ACT. Loss or modification of the target antigen has not only been identified in ACT, but also in other immunotherapy modalities [206, 207]. Loss of the target molecules could be secondary to alternative slicing [208] or interruption of antigen presentation to the cell surface [209]. Furthermore, a diminishment of target molecule density on the cell surface can lead to evasion of CAR-T cell therapy [210]. Low or loss of expression of target molecules on tumor cells at relapse can be secondary to pre-existing malignant cell heterogeneity [211] or lineage switching [212].

### Novel construction and combination strategies to improve ACT efficacy

#### Novel design of CAR-T cells

Development of universal CAR-T cells is one approach to address the intrinsic defects of T cells from patients with hematological malignancies. To improve the proliferation of CAR-T cells, inclusion of a stronger co-stimulatory domain, such as 4-1BB instead of CD28, or incorporation of both 4-1BB and CD28 (the third-generation CAR-T cells), can improve the persistence of CAR-T cells [213, 214]. To alter the immunosuppressive TME, a fourth generation of CAR-T cells called TRUCKs (“T cells redirected for antigen‐unrestricted cytokine‐initiated killing”) has been designed and has entered early phase clinical trials. TRUCKs have a transgene, usually immunostimulatory cytokines, under the control of the NFAT‐responsive/IL‐2 minimal promoter. CAR engagement and activation leads to NFAT phosphorylation and transgene expression that acts in an autocrine fashion to stimulate CAR-T cells, or paracrine to modulate the immune cell environment [215]. Other than optimizing the CAR-T cell design, an alternative strategy is to combine ACT with another therapy and maximize the antitumor activity (Table 6).

**Table 6:** Combination strategies to enhance the efficacy of adoptive cell therapy

Category	Example	Mechanisms
Combination with negative immune regulator blockade	Immune checkpoint inhibitor	Remove the suppression of CAR cell function through the checkpoint pathway
	Knockout TGF-β signaling in CAR cells	Enhance CAR cell function and alter TME
Combination with lymphodepletion	Fludarabine and cyclophosphamide	Suppress immune response and elimination of CAR cells
		Deplete T_reg_ and other competing immune cells
CAR T cell combination	Two CAR cells targeting the same molecule	Overcome immune elimination of the first CAR cells
	Two CAR cells targeting the different molecules	Maximize therapeutic effects and reduce antigen escape
Combination with immune modulators	Exogenous immune modulators	Stimulate CAR cells and other cytotoxic immune cells and reduce immunosuppressive cells
	Fourth-generation CAR T cells	Deliver immune regulators at cancer sites
Combination with TKI	CAR T cells with ibrutinib	Exert direct antitumor activity, downregulate PD1/PD-L1, tilt from Th2 to Th1, suppress MDSC, etc
Combination with oncolytic virotherapy	Armed oncolytic adenovirus	Exert direct lysis and killing of cancer cells, stimulate innate immune response, alter TME, cytokine to attract and stimulate CAR T cells

#### Combination of ACT with immune checkpoint inhibitors

PD1 is upregulated after CAR-T cell infusion which can down-regulate the CD28 co-stimulatory signaling and induce the CAR-T cell dysfunction [205]. Both preclinical and several clinical trials suggest that the PD1/PD-L1 blockade and CAR-T combination therapies can achieve synergistic anti-tumor activity [216–218]. To eliminate the negative effects of PD-1/PD-L1 axis on the function of CAR-T cells, CAR-T cells have been modified with the knockdown of the PD1-encoding gene PDCD1 [219]. These PD1-deficient CAR-T cells possess increased antitumor activity similar to the combination of CAR-T cells and anti-PD1 antibodies without the systemic toxicity. In addition to PD1/PD-L1, inhibition of other immunosuppressive pathways has also been explored in ADT. For example, as discussed above, TGF-β is a major immunosuppressive regulator affecting multiple immune cells in the TME. Knockout of the TGF- β signaling in CAR-T cells enhances CAR-T cell proliferation and augments antitumor activity [220].

#### Combination with lymphodepletion

After CAR-T therapy, relapse with malignancies carrying the target antigen represents a potential opportunity to re-treat with the same CAR-T cell therapy. However, in many cases, a re-challenge with the same CAR-T cells frequently fails to induce a response [206]. One mechanism of resistance is that patients have developed an immune response to the single-chain variable fragment of CAR that can eliminate re-infused CAR-T cells. To prevent the development of immune response to the CAR, intensified lymphodepletion before CAR-T therapy has been used with improved clinical activities [221, 222]. Furthermore, lymphodepletion can minimize the effects of regulatory T cells, reduce other immune cells that compete for homeostatic cytokines and enhance APC activation.

#### Combination of two different CAR-T cell therapies

Instead of using lymphodepletion to prevent immune response and elimination of CAR-T cells, an alternative strategy is to combine two types of CAR-T cells with different structures of CARs targeting the same target molecules. In this case, the second type of CAR-T cells can survive and kill tumor cells even after the recipient patients develop immune response to the first CAR-T cells. It has been shown that humanized CD19 CAR-T therapy can overcome immune-mediated rejection of murine-derived anti-CD19 CAR-T therapy [223].

Another ACT combination strategy is to use CAR-T cells targeting two different antigens on tumor cells. One mechanism of ACT failure is tumor cell heterogeneity wherein some tumor cells do not express the target molecule. Targeting two different molecules on the same malignant cell can maximize tumor cell killing and decrease recurrence. This can be achieved through a tandem construct of a single CAR vector that targets two different antigens [224] or a combination of two different types of CAR-T cells, each targeting different antigens [210].

#### Combination with immune modulators

In order to create an immunostimulatory milieu, CAR-T cells can be combined with other immunostimulatory molecules, such as immunostimulatory cytokines, cytokine receptors and co-stimulatory molecules, or even modified to directly express these molecules as seen in the fourth generation of CAR-T cells [225]. IL-12 is one of the cytokines that has been extensively studied in preclinical models. It can enhance the cytotoxic activity of CD8 + T cells and NK cells, stimulate the Th1 helper T cell response and counteract the immunosuppression by T_reg_ and MDSCs. A clinical trial with the fourth generation of CAR-T TRUCK cells expressing IL-12 has been initiated [226].

#### Combination with small molecule inhibitors

In addition to its direct antitumor activities, ibrutinib, a BTK inhibitor, has several other immunomodulatory effects. It can downregulate PD1 expression in CD4+ and CD8 + T cells, PD-L1 expression on CLL-affected B-cells and IL-10 production [227]. Furthermore, ibrutinib is an irreversible ITK inhibitor which leads to a preferential inhibition of Th2 in favor of the Th1 differentiation [113], and conversion of MDSCs to dendritic cells [228]. Currently, several clinical trials with the combination of ibrutinib and CAR-T cell therapy are ongoing. In addition to ibrutinib, several other tyrosine kinase inhibitors, such as EGFR inhibitors, are being explored in combination regimens.

#### Combination with oncolytic virotherapy (OV)

Several preclinical studies on the antitumor activity of oncolytic virotherapy with ACT have already been reported [229]. With this combination, oncolytic viruses can target and kill cancer cells, stimulate an innate immune response and create a stimulatory immune milieu to potentiate ACT. Because OVs are usually cancer-specific, they can express transgenes on the surface of cancer cells which can then be recognized by CAR-T cells. Furthermore, armed OVs can express cytokines that can attract CAR-T cells to the tumor sites and enhance the cytotoxicity of CAR-T cells [230]. So far, this combination has yet to be translated into clinical applications.

## Virotherapy and innate immune modifiers

### Innate immune system

#### Overview of the innate immune system

The innate immune system is the first line of defense against infections and foreign substances. In addition to anatomical barriers, it consists of different cells involved in broad-spectrum pattern-based recognition and response to foreign substances. It is not only an obligate prerequisite for the induction of adaptive immune response, but also a prerequisite of effective ways of clearing foreign substance, including killing tumor cells independent of T cells. Like adaptive immune response, the innate immune system is also tightly regulated through built-in stimulatory and inhibitory feedback networks which are being exploited for cancer immunotherapy. Considering the overall disappointing efficacy of ICBs, innate immune cells and their regulatory molecules represent attractive alternatives for improving and/or complementing ICBs for cancer therapy (Table 7). Natural killer cells and phagocytes, including macrophages and dendritic cells, are commonly studied for cancer immunotherapy [165, 169, 192, 231].

**Table 7:** Innate immune cells for cancer immunotherapy

Natural killer cells	Stimulatory	Activating receptors: NKP30, NKP44, NKG2D, CD16, 2B4
		Cytokines: IL-2 and IL-15
		Engineered NK cells
	Inhibitory	ITIM-containing receptors (KIR family, PD1 and TIGIT)
		CD94/NKG2A
		LIR1
		TIM-3
		IDO
Dendritic cells	Stimulatory	cGAS-STING pathway
		TLR
		Cytokines: GM-CSF, Type I IFN, FLT3L
	Inhibitory	ITIM-containing receptors (FcγRIIB, etc.)
		CD39/CD73/adenosine pathway
		IDO
Macrophages	Stimulatory	CpG oligonucleotide
		IFN-γ
		TNF-α
		IL-12
		TLR agonists
	Inhibitory	ITIM-containing receptors (SIRPα-CD47, SIGLEC-10, etc.)
		IL-10, IL-4, IL-13
		CCL5, CCL2/CCR2

#### Enhancing the innate immune system for cancer immunotherapy

NK cells can be stimulated through the activating receptors, such as NKP46, NKP30, NKP44, NKG2D, CD16 and 2B4, and via cytokines, such as IL-2 and IL-15 [232]. Activation of NK cells can directly contribute to anti-cancer immunity through their direct cytotoxic activities, attraction of dendritic cells and cytokine production, such as IFN-γ. IL-2 can not only stimulate cytotoxic T cells, but also expand NK cells. ALT-803 includes an IL-15 mutant (IL-15N72D) and a dimeric IL-15 receptor α sushi domain-IgG1 Fc fusion protein. It acts as an IL-15 super-agonist [233]. Because of the critical roles that NK cells also play in adaptive immune response, clinical trials are currently ongoing not only for ALT-803 alone, but also in combination with ICBs (clinicaltrials.gov ID: NCT02523469, NCT03228667 and NCT03853317).

Dendritic cells play critical roles in antigen presentation, cross-priming and activation of cytotoxic T cells, and produce CXCL9 and CXCL10 to recruit tumor-specific T cells to the tumor sites. During immune response, NK cells release CC-chemokine ligand 5 (CCL5), XC-chemokine ligand 1 (XCL1), XCL2 and FLT3LG, which recruit dendritic cells to stimulate the immune response [234]. Dendritic cells are strongly activated by type I interferons which are induced upon activation of specific pattern-recognition receptors, such as toll-like receptor (TLR) 3, TLR 7 and TLR 9. Multiple drugs targeting TLRs have already reached the clinical trial stage in combination with standard of care or ICBs [235]. In one study, a TLR3 agonist poly-ICLC was combined with Fms-like tyrosine kinase 3 ligand (Flt3L) and localized radiotherapy in patients with indolent non-Hodgkin’s B cell lymphoma. FLT3L promotes the commitment of hematopoietic progenitor to the dendritic cell lineage, recruits intratumoral dendritic cells and enhances dendritic cell survival and proliferation. As discussed above, local radiotherapy converts cancer cells into an in situ vaccine while poly-ICLC activates dendritic cells. This combination increases dendritic cell number and activation at tumor sites and induces abscopal effects [236].

Type I IFN expression can be induced by the cGAS-STING pathway which plays critical roles in the innate immune system [237–239]. Upon binding to cytosolic DNA, cGAS triggers the reaction of GTP and ATP to form cyclic GMP-AMP which binds to STING. In addition to induction of Type I IFN expression, activation of the cGAS-STING pathway upregulates the expression of NK cell receptor NKG2D ligands that can lead to the recognition and destruction of cancer cells by NK cells and T cells. Because of the important roles of the cGAS-STING pathway in stimulating the anti-cancer immunity, several STING agonists have entered into clinical trials with the combination of ICBs to improve their clinical efficacy [240].

Macrophages are professional phagocytes that can internalize large particles, such as debris, apoptotic cells and pathogens, to maintain homeostasis in the human body. According to their functions, TAM can be classified into two major subsets: classically activated inflammatory macrophages (M1, CD80 + and CD86 +) that promote anti-cancer immunity and alternatively activated anti‐inflammatory macrophages (M2, CD 68 +, CD163 + and CD206 +) that are immunosuppressive. Given the key roles played by macrophages, several cytokines (such as IL-12), immunoagonists and inhibitors of TAMs are being explored to polarize and tilt macrophages to the M1 phenotype. For example, CpG oligonucleotide, which is also a TLR9 agonist, can not only induce macrophage polarization to the M1 phenotype [241], but also induce memory T cells and abscopal anti-tumor activity [242].

#### Suppression of the negative regulators of the innate immune system

Like the adaptive immune response, the innate immune system has many negative regulators that can be targeted to boost anti-cancer immunity. The immunoreceptor tyrosine-based inhibition motif (ITIM) is a conserved amino acid sequence found in the cytoplasmic tails of many inhibitory receptors on immune cells [243]. Some of the ITIM-containing receptors, such as PD1, has already been targeted for cancer therapy. The killer-cell immunoglobulin-like receptors (KIRs) are a family of ITIM-containing receptors identified from the seminal discoveries in 1990s by Alessandro Moretta et al. [244]. They are expressed on the plasma membrane of NK cells and some T cells. Most KIRs are inhibitory in that their recognition of MHC molecules suppresses the cytotoxic activity of NK cells [245]. Under normal conditions, these KIRs recognize autologous cells and prevent auto-reactive cytotoxicity which can also dampen the cytotoxicity of NK cells against HLA-expressing cancer cells. An anti-pan-KIR2D agent, lirilumab, has already reached clinical trials. The combination of lirilumab and nivolumab is well tolerated and showed promising clinical activities with an overall response of 76% (16/21) in relapsed/refractory classical Hodgkin lymphoma [246]. Other ITIM-containing receptors are being extensively studied [247]. For example, LIR-1 (binding to HLA-G and other low affinity HLA ligands) is expressed on approximately one-third of NK cells. An antibody blocking LIR-1 significantly potentiated the tumoricidal activity of NK cells in vitro and in vivo [248].

In addition to KIRs, other MHC-recognizing receptors are also important in regulating NK cell functions and tumor eradication. NKG2A (binding to HLA-E) is constitutively expressed in approximately 50% NK cells and can be induced in T cells upon cytokine stimulation or antigen-induced activation [249]. Preclinical studies showed that an anti-NKG2A antibody had remarkable antitumor effects and synergized with the anti-PD1 antibody durvalumab in unleashing NK and CD8 + T cell function [250]. Promising results were shown in a Phase II trial of the humanized anti-NKG2A antibody monalizumab in combination with cetuximab with a 31% objective response rate in patients with previously treated head and neck cancers [250].

Since most of the TAMs are M2 macrophages, several approaches have been explored to inhibit or deplete TAMs for cancer immunotherapy. Several cytokines, such as CCL5 and CCL2/CCR2, are involved in recruiting macrophages to the tumor sites and promoting cancer growth. Several preclinical studies showed that tumor growth inhibition could be achieved through targeting these cytokines or with depletion of TAMs. However, they have yet to be translated into clinical applications as these cytokines have other functions in addition to recruiting macrophages [251].

CD47 is a transmembrane protein expressed in all types of cells, serves as a self‐marker and interacts with signal regulatory protein α (SIRPα) to inhibit phagocytosis by immune cells [252]. SIRPα is a transmembrane protein expressed on macrophages, granulocytes, monocytes, dendritic cells and neurons. The recognition of CD47 by SIRPα generates a “don't eat me” signal and has been used by cancer cells to evade anti-cancer immunity. Many inhibitors have been developed to block the CD47‐SIRPα pathway and enhance tumor immunotherapy, including anti‐CD47 therapy and anti‐SIRPα therapy [253]. An early clinical trial showed that the combination of an anti-CD47 blocking antibody Hu5F9-G4 and the CD20 antibody rituximab had promising clinical activities with an objective response rate of 40% with relapsed or refractory diffuse large B-cell lymphoma and 71% with follicular lymphoma [254].

### Virotherapy

#### Overview

Virotherapy uses viruses to target and kill cancer cells, induce innate and adaptive immune response for cancer treatment. Adenoviruses, herpes viruses, measles viruses, coxsackie viruses, polioviruses, reoviruses, poxviruses and Newcastle disease viruses, among others, are some of the oncolytic viruses (OVs) under preclinical and clinical development for cancer therapy [255]. Initially wild type viruses were used. Because those viruses are potentially associated with adverse events caused by viral replication in normal cells, almost all OVs under current development are modified with strong cancer tropism and usually armed with transgenes to enhance the efficacy and/or potentiate combination therapy. So far, three OVs have been approved and used at clinic: Rigvir (an oncolytic picornavirus) [256], H101 or Oncorine (an adenovirus) [257] and talimogene laherparepvec, also known as T-vec (a herpes simplex-1 virus encoding for GM-CSF) [179].

#### Mechanisms of action

Several mechanisms have been proposed for the action of OVs. First, OVs can directly target, lyse and kill cancer cells. Cancer tropism can occur naturally with OVs as oncogenic signaling pathways are more active in cancer cells which can facilitate viral replication. More recently, genetic engineering has been used to make cancer-specific OVs. For example, mutations/deletions can be introduced in genes that are required for replication in normal but not in cancer cells; critical viral genes are under the control of cell-specific promoters active in cancer cells, but not in normal cells; and expression of cancer-targeting viral receptors can guide cancer-specific tropism [258]. Second, OVs can activate the innate immune response. Viral replication and destruction of tumor cells and release of viral DNA can induce innate immune response locally to kill more cancer cells. Third, OVs can induce adaptive immune response. Viral replication and destruction of tumor cells alter TME, trigger chemotaxis and accumulation of cytotoxic lymphocytes to the site of infection, and can convert immune “cold” to “hot” tumors; viral replication induces ICD, and releases DAMPs, such as calreticulin, high-mobility group protein B1 (HMGB1) and ATP, along with tumor-associated antigens. Fourth, OVs can be engineered to express transgenes to stimulate immune response. For example, talimogene laherparepvec expresses GM-CSF and was FDA approved for treatment of recurrent melanoma. Several other immunoregulatory genes are currently being explored for their potentiation to stimulate immunotherapy with OVs, such as IL-2, IL-12, IFN-α/β, 4-1BB and CD40L [259].

#### OVs armed with transgenes

Even though oncolytic virotherapy showed promising preclinical activities, clinical activities are still moderate. In cutaneous melanoma, intratumoral injection of talimogene laherparepvec is associated with an overall response rate of 26.4% [179]. One advantage of OVs is that they can be armed with transgenes and combined with several therapeutic interventions. So far transgenes targeting every step along the anti-cancer immunity cycle have been studied at least in preclinical models (Table 7) [260]. For example, oncolytic adenovirus armed with bi-specific T cell engager (BiTE) has been demonstrated to induce both oncolysis by OV and engagement and activation of cytotoxic T cells which led to immune-mediated destruction of cancer cells both in vivo with tumor implants and in primary ex vivo patient specimens [261, 262].

#### Combination therapies with OVs in clinical development

While most OVs armed with the transgenes listed on Table 8 are still at the preclinical stages, several clinical trials combining OVs with another therapeutic agent have been initiated [263]. The most common combination therapy is OVs and ICBs. These two agents have complementary mechanisms of anti-cancer immunity in that ICBs release the suppression of anti-cancer immune response while OVs stimulate the immune response. The combination was first tested in preclinical models with an oncolytic Newcastle disease virus (NDV) in combination with systemic CTLA-4 blockade [264]. Local virotherapy induced abscopal effects and immune cell infiltration at distant tumors.

**Table 8:** Strategies to arm oncolytic viruses for cancer immunotherapy

The anti-cancer immunity cycle	Transgenes to arm OVs and enhance cancer immunotherapy	Example transgenes
Step 1. Cancer cell death and antigen release	Molecules inducing immunogenic cell death	Type I IFN, TNFα, TRAIL
Step 2. Antigen presentation	Tumor-associated antigens, cancer vaccine, chemokine	Truncated CD19, cancer vaccine, GM-CSF
Step 3. Priming and activation	Checkpoint inhibitors, co-stimulatory molecules, immunostimulatory cytokines	Anti-CTLA4 miniantibody, anti-PD1, IL-2, IL15, OX40L, 4-1BBL
Step 4. T cell trafficking	Molecules targeting tumor vasculature,	VEGF/VEGFR inhibitor, endostatin,
Step 5. Infiltration into tumors	Chemokines to attract T cells; molecules targeting tumor stroma and matrix degradation	CXCL9, CXCL10 CXCL11, CCL2, CCL5, hyaluronidase, collagenase, MMP-9
Step 6. Recognition of tumor cells by T cells	Bi-specific T cell engager (BiTE)	BiTE targeting CD3 and CD19, BiTE targeting EpCAM and CD3
Step 7. Killing of cancer cells	Checkpoint inhibitors, co-stimulatory molecules, immunostimulatory cytokines, molecules targeting TME metabolism, molecules targeting or depleting inhibitory immune cells	Anti-CTLA4 miniantibody, anti-PD1, IL-2, IL15, OX40L, 4-1BBL, CD39/CD73/A2aR, IDO inhibitors

Since then, many trials combining OVs with anti-CTLA and anti-PD1/PD-L1 antibodies either have already been completed or are in progress to determine the efficacy and toxicity of the combinations. In one study with unresectable stage IIIB–IV melanoma, the combination of ipilimumab and talimogene laherparepvec significantly improved the objective response rate to 39% from 18% with ipilimumab alone (OR 2.9, 95% CI 1.5–5.5, *P* = 0.002), and induced abscopal effects on visceral lesions [265]. In addition to talimogene laherparepvec, several other major OVs are being combined with ICBs in clinical trials.

OVs have also been tested in clinical trials with chemotherapy. In a Phase I/II trial of carboplatin/paclitaxel plus reovirus, the combination therapy was well tolerated with significant clinical activity and minimal viral toxicity [266]. Another Phase I trial determined the toxicity of the combination of gemcitabine with replication-competent adenovirus Ad5-yCD/mutTK(SR39)rep-ADP (Ad5-DS) in locally advanced pancreatic cancer. This adenovirus expressed double-suicide genes: yeast cytosine deaminase (yCD) and herpes simplex virus 1 thymidine kinase (HSV-1 TK) [267]. This combination was well tolerated in early clinical trials.

## Therapeutic cancer vaccine

Cancer vaccine is a targeted cancer immunotherapy that uses putative cancer antigen(s) or antigenic epitope(s) presented in protein, RNA, DNA, viral or bacterial vectors, cells or other means to stimulate anti-cancer immunity. In contrast to vaccination for infectious diseases in general, the efficacy of early cancer vaccines is disappointing, with objective response rates less than 5% [268]. More recently, with the understanding of anti-cancer immunity and development of vaccine technology, deep sequencing and bioinformatics, personalized cancer vaccines showed promising clinical activities. However, many patients do not respond. The reason for low efficacy for cancer vaccines is likely to be multifactorial. Cancer vaccines are used in patients whose immune system has already tolerated cancer and cancers can develop or have already developed an immunosuppressive TME that prevents anti-cancer immunity. In contrast, vaccines for infectious diseases are exogenous antigens that hosts have not developed resistance.

### Cancer vaccine antigens

There are two major types of cancer vaccines: shared tumor-associated antigens (TAA) and unique tumor antigens (Table 9). Shared TAAs include cancer/testis antigens, cell differentiation antigens and overexpressed cell antigens. Cancer/testis antigens are a group of proteins, such as cancer/testis antigen 1 (CTAG1B, often referred to New York esophageal squamous cell carcinoma 1 or NY-ESO-1), melanoma-associated antigen 1 (MAGE-A1), MAGE-A3, etc., that are normally expressed in immune privileged germline cells, but upregulated in some cancer cells. Cell differentiation antigens are a group of antigens expressed in differentiated tissues from which some cancers develop and share those antigens, such as glycoprotein 100 (gp100), prostatic acid phosphate, and prostate-specific antigen (PSA). Overexpressed cell antigens are expressed in normal cells, but significantly upregulated in some cancer cells, such as mucin 1 (MUC1) or epithelial membrane antigen, mesothelin and HER2. There are two major issues associated with shared TAAs. First, the immune system has already developed tolerance to these antigens. Hence, even with strong adjuvants, co-stimulators or repeated vaccinations, anti-cancer immunity may develop, but is not sufficient to eliminate cancers. Second, these antigens are also expressed in some normal cells. Hence, untoward damage may develop against those normal cells/tissues expressing the target antigens.

**Table 9:** Classification of cancer vaccine antigens

Category	subcategory	Tumor specificity	Immune tolerance	Prevalence	Potential for cancer vaccine
Cancer-associated antigens	Cancer/testis antigens	Usually high	Low	Intermediate	Intermediate
	Overexpressed and differentiation antigens	Variable, but usually low	High	High	Low
Cancer-unique antigens	Cancer viral antigens	High	Low	Intermediate	High
	Neoantigens	High	Low	Low and usually unique	High

Unique tumor antigens include oncogenic viral antigens associated with viral infection and tumor neoantigens associated with cancer genomic alterations, such as mutations, frame shift and gene fusion. The three most commonly studied viruses associated with cancers and cancer vaccines are hepatitis B virus, human papillomavirus (HPV) and Epstein–Barr virus (EBV). Because these unique tumor antigens appear after the immune system has already developed, the central tolerance to these antigens and cross-reactivity to normal tissues are usually low and the likelihood of induction of immune response to them is high.

### Vaccine delivery vehicles

Several approaches have been used in clinic to deliver cancer antigens in order to elicit anti-cancer immunity (Table 9). Cell-based vaccines use autologous cancer cells, antigen-presenting cells or allogeneic cells to deliver cancer vaccine antigens. When cancer cells are used, cells are treated in vitro, usually with radiation, to prevent further cell division before administration. The advantage of cell-based vaccine is that specific target antigens do not need to be prospectively identified, and dendritic cells can present tumor antigens in the context of the MHC that can be further potentiated with a stimulatory cytokine. Sipuleucel-T is FDA approved for treatment of metastatic prostate cancer and is comprised of autologous dendritic cells activated ex vivo with expression of a chimeric protein of immune stimulatory cytokine, GM-CSF, fused to a cell differentiation tumor antigen PAP. In a Phase III clinical trial, the median overall survival (OS) of the sipuleucel-T group was about 4 months longer than the control cohort treated with placebo [269]. However, the vaccine treatment was not associated with any biomarker (PSA) or radiological response. In fact, subgroup analyses showed there was no OS difference between the therapeutic and control groups of young patients (< 65 years) who were supposed to have more robust immune response [270]. Several other cell-based vaccines are also at clinical trials. Both gemogenovatucel-T and GVAX are tumor cell vaccines expressing GM-CSF to promote antigen presentation, activation and survival of dendritic cells. Some other cellular vaccines are manipulated to down-regulate the expression of immunosuppressive factors. For example, belagenpumatucel-L is a mixture of four irradiated human NSCLC cell lines, transfected with a TGF-β2 antisense gene. A higher response rate was observed in patients with NSCLC treated with higher doses of Belagenpumatucel-L [271]. However, a Phase III trial failed to demonstrate the OS benefit when it was used as a maintenance therapy after platinum-based chemotherapy in Stage III/IV non-small cell lung cancer [272]. Gemogenovatucel-T expresses a bi-functional short hairpin RNA to knock down the expression of the enzyme furin which converts immunosuppressive TGF-β1 and TGF-β2 into active isoforms. Even though those cell-based vaccines showed promising activities in preclinical models and stimulated immune response in patients, their clinical efficacy has yet to be proven [273].

In addition to human cells, microorganisms such as bacteria and yeasts are also being explored for cancer vaccine therapy. Heated-inactivated bacteria and Bacillus Calmette-Guérin have been used in clinic to treat cancers for decades. But strictly, they are not cancer vaccines as they do not carry tumor antigens. Other bacteria, such as Listeria, can deliver DNA- and RNA-encoded tumor antigens directly into mammalian cells, including APCs, and are being tested as cancer vaccine vehicles [274].

For microorganisms, viral vectors have been studied as a vehicle to deliver cancer vaccines. For viral vectors, in addition to its ability to induce innate and adaptive immune response, exogenous genes can be incorporated and expressed, including cytokines and tumor antigens. One potential disadvantage of using a viral vector to deliver a cancer vaccine is that the immune response induced by previous vaccinations or infections of the same virus produces a neutralizing antibody and clears subsequent vaccinations. One approach is to administer the vaccine through intratumoral injection as talimogene laherparepvec. Another approach is to use a heterologous prime/boost strategy which cancer antigens are delivered by two different viral vectors at the primary and boost vaccinations (Table 10).

**Table 10:** Major vehicles to deliver cancer vaccines

Technology	Examples	Important considerations
Peptides	Short peptide (< 15 aa)	Directly binding to MHC, Not processed by APC, more tolerogenic,
	Synthetic long peptide, neoantigens	Preferably taken up and processed by dendritic cells, usually co-administrated with adjuvant to potentiate immunogenicity
Cellular vaccine	Tumor cells	Autologous or allogeneic, not need to identify tumor antigens,
	Dendritic cells	Provide tumor antigens and costimulatory signals, can co-express cytokines and other co-stimulatory molecules, highly immunogenic
	Microorganisms	Microorganisms are immunostimulatory, can co-express other stimulatory molecules
Viral vector	PROSTVAC-VF/Tricom	Vehicles are highly immunogenic, can co-express stimulatory cytokines and other molecules; may need local injection; neutralizing antibody can clear virus
DNA/RNA	RNA mutanome vaccines	Vaccine (DNA/RNA) itself is immunogenic. Low delivery efficiency with the native form. Other delivery methods (nanoparticles, gene gun and in situ electroporation) enhance delivery

Peptide vaccines have also been used in vaccine studies as T cells recognize tumor antigens presented by MHC. When short peptides are used, they can directly bind to MHC molecules on any nucleated cells. As T cell activation requires the engagement of T cell receptors by antigens presented with MHC as well as a second signal from a co-stimulatory molecule, presentation of short peptide vaccines by nucleated cells other than APCs often induces T cell anergy and immune tolerance since other nucleated cells lack co-stimulatory signals. To overcome the immune tolerance associated with short peptides, synthetic long peptides (SLPs) have been used. SLPs are preferentially taken up and processed by dendritic cells which also provide co-stimulatory molecules to prime and activate T cell immunity. To further improve the efficacy, SLPs are often formulated with inflammatory adjuvants. For example, synthetic cancer neoantigen long peptide vaccines formulated with a toll-like receptor 3 (TLR3) ligand poly-ICLC (polyinosinic and polycytidylic acid) induced strong anti-cancer immunity and clinical response [275]. In this study, vaccination stimulated polyclonal neoantigen-specific CD4 + and CD8 + T cell responses and immune cell migration into intracranial glioblastoma tumors. Systemic immune response was abolished in those patients who received the immunosuppressive steroid dexamethasone during vaccine priming.

Peptides and proteins have also been formulated in nanoparticles to enhance its efficacy. Nanoparticles can protect peptides/proteins from degradation, prolong circulation time, control antigen release, confer targeted delivery through decorating cancer targeting peptides on the surface[276–278], and formulate with adjuvant agents to enhance immune response [279, 280]. Furthermore, by optimizing the structure, surface charge and cell penetrating peptides, the efficacy of nanoparticles can possibly be further enhanced [281, 282]. So far, various nanomaterials, such as polymeric materials, liposomes, micelles, silica nanoparticles, gold nanoparticles (AuNPs) and virus nanoparticles, have been studied in preclinical models with a few translated into clinical trials, but the efficacy is still yet to be validated [279].

Instead of using peptides and proteins, DNA and RNA vaccines have also been translated into clinical trials [283]. After administration, DNA and RNA can be taken up by antigen presenting cells that then translate into peptide/proteins and present to immune cells. In addition to serving as vaccines, exogenous DNA and RNA can serve as immune stimulators, trigger nucleic acid sensors and activate dendritic cells through certain TLR and STING pathways. One major disadvantage of naked DNA and RNA vaccine is their low delivery efficiency. To overcome this obstacle, various delivery methods have been developed, such as viral vectors and nanoparticles as discussed above, gene gun, electroporation and so on [284]. Two coronavirus (COVID-19) mRNA vaccines approved in the US are both RNA vaccines formulated in lipid nanoparticles [285, 286]. For cancer vaccines, RNA lipid nanoparticles, either alone or in combination with ICBs, induced durable clinical responses, associated with strong CD4 + and CD8 + T cell immunity against the vaccine antigens [287].

### Combination therapy with cancer vaccines

Cancer vaccines are designed to bypass and/or stimulate the first three steps of the anti-cancer immunity cycle: cancer antigen release and presentation, immune cell priming and activation of immune cells. Once immune cells are activated, they still need to go through the remaining four steps along the cycle: mobilize in the periphery, infiltrate into cancer sites, recognize cancer cells and elicit cytotoxicity toward cancer cells. Hence, resistance mechanisms governing the anti-cancer immunity, especially those in the TME, can still dampen the efficiency of cancer vaccines and are being explored to potentiate cancer vaccines.

The most critical function of cancer vaccines is to present cancer antigens to prime and activate T cells and induce anti-cancer immunity. In many cancers, little or no anti-cancer immunity exists in patients that manifests as cold tumors with little immune cell infiltration at the tumor sites. These cold tumors usually do not respond to ICBs, as there is no ammunition to fire at cancer cells upon removal of the brake by ICBs. To improve their efficacy, cancer vaccines have been extensively studied to be combined with adjuvant agents, such as a TLR-3 agonist poly-ICLC, to stimulate immune response. Immune response to the cancer/testis antigen NY-ESO-1 is higher when NY-ESO-1 vaccine is combined with poly-ICLC [288].

Many trials are currently ongoing to determine the efficacy and toxicity of cancer vaccines in combination with cytokines. For example, IL-2 plays critical roles in key functions of immune response. Compared to IL-2 alone, the combination of IL-2 with a tumor-associated antigen gp100 significantly improved the overall response rate (ORR: 16% vs. 6%, *p* = 0.03) and progression-free survival (PFS: 2.2 versus 1.6 months, *p* = 0.008) [289]. In addition to IL-2, several other immunostimulatory cytokines are being explored. IL-12 is a multipotent cytokine that stimulates T and NK cells, regulates other cytokines and multiple aspects of immune response. Several clinical trials are currently ongoing to determine the efficacy and toxicity of IL-12 and cancer vaccine combinations.

Extensive preclinical studies as well as many clinical trials are currently ongoing to combine cancer vaccines with ICBs. GX-188E (tirvalimogene teraplasmid) is a therapeutic HPV DNA vaccine that encodes HPV-16 and HPV-18 E6 and E7 [290]. When GX-188E was combined with pembrolizumab in HPV-16/18-positive advanced cervical cancer, 11 of 26 patients (42%; 95% CI 23–63%) achieved a response; four patients (15%) had a CR at 24 weeks. This combination was well tolerated [291]. Similar results were observed in another Phase II trial with a synthetic long-peptide HPV-16 vaccine ISA101 combined with nivolumab in HPV-16-positive solid tumors; this combination showed a 33% ORR and 17.5-month median OS [292]. Other than concurrent use, ICB has also been shown to have activity as a salvage therapy after cancer vaccine failure. Ott et al. showed that pembrolizumab induced complete responses in melanoma patients after failure of treatment with synthetic long peptide vaccine of tumor neoantigens with poly-ICLC adjuvant [293]. With a follow-up of almost four years, long-term persistence of neoantigen-specific T cells was still observed with the development of memory T cell phenotype, tumor infiltration and epitope spreading [294].

In addition to multiple studies focusing on anti-PD1/PD-L1 and anti-CTLA4 antibodies, other immune co-inhibitory and co-stimulatory agents are being actively studied to enhance the efficacy of cancer vaccines. One Phase I clinical trial has already been reported with a melanoma-associated antigen recognized by T cells 1 (MART-1) peptide vaccine plus IMP321, a fusion protein consisting of four LAG-3 extracellular Ig-like domains fused to the Fc fraction of a human IgG1 (LAG-3Ig). The combination group is associated with significant increase of MART-1-specific CD8 T cells (*p* < 0.02), higher proportion of CCR7^−^ CD45RA^+/−^ CD8 T effector cells (*p* < 0.02) and reduced expansion of regulatory T cells (*p* < 0.04) [295].

## Conclusion remarks

A few combination therapies have been approved by the FDA to improve clinical efficacy of ICIs. With increasing research in identifying action-driven reliable biomarkers in guiding clinical immuno-oncology decisions, IO combinations among ACT, novel ICIs, cancer vaccines and small molecule inhibitors are expected. In this regard, the future of cancer immunotherapy awaits for a truly patient-oriented, individualized approach.

## Data Availability

The material supporting the conclusion of this review has been included within the article.

## Abbreviations

ACT: adoptive cell therapy
ADT: androgen deprivation therapy
APC: antigen presenting cell
ATP: adenosine triphosphate
B-ALL: B-cell acute lymphoblastic leukemia
BiTE: bi-specific T cell engager
BTK: Bruton's tyrosine kinase
CAF: cancer-associated fibroblasts
CAHON: Chinese American Hematologist and Oncologist Network
CAR: chimeric antigen receptor
CCL: C–C motif chemokine ligand
CD: cluster of differentiation
CDK: cyclin-dependent kinase
cGAS: cyclic GMP-AMP synthase
CLL: chronic lymphocytic leukemia/lymphoma
CTLA4: cytotoxic T-lymphocyte-associated protein 4
CXCL: C-X-C motif chemokine ligand
DAMP: damage-associated molecular pattern
dMMR: defective DNA mismatch repair system
DNA: deoxyribonucleic acid
DNMT: DNA methyltransferase
EBV: Epstein–Barr virus
EGFR: epidermal growth factor receptor
EMT: epithelial–mesenchymal transition
FDA: Food and Drug Administration
GM-CSF: granulocyte–macrophage colony-stimulating factor
HDAC: histone deacetylase
HLA: human leukocyte antigen
HMGB1: high-mobility group protein B1
HPV: human papilloma virus
HSP: heat shock protein
ICB: immune checkpoint blocker
ICD: immunogenic cell death
IDO: indolamine-2,3-dioxygenase
IFN: interferon
IL-2: interleukin-2
IO: immuno-oncology
ITIM: immunoreceptor tyrosine-based inhibition motif
ITK: IL-2-inducible T cell kinase
KIR: killer-cell immunoglobulin-like receptor
LAG-3: lymphocyte-activation gene 3
MAGE-A1: melanoma-associated antigen 1
MART-1: melanoma-associated antigen recognized by T cells 1
MDSC: myeloid-derived suppressor cell
MEK: mitogen-activated extracellular kinase
MHC: major histocompatibility complex
MSI-H: microsatellite instability high
NDV: Newcastle disease virus
NK: natural killer
NMPA: China National Medical Product Administration
NSCLC: non-small cell lung cancer
NY-ESO-1: New York esophageal squamous cell carcinoma 1
ORR: overall response rate
OS: overall survival
OV: oncolytic virotherapy
PD1: programmed cell death 1
PDGFR: platelet-derived growth factor receptor
PD-L1: programmed cell death ligand 1
PEG: polyethylene glycol
PFS: progression-free survival
PMN-MDSC: polymorphonuclear myeloid-derived suppressor cell
poly-ICLC: polyinosinic and polycytidylic acid
PSA: prostate-specific antigen
ROS: reactive oxygen species
RT: radiotherapy
SCLC: small cell lung cancer
SIRPα: signal regulatory protein α
STING: stimulator of interferon genes
TAM: tumor-associated macrophage
TCR: antigen-T cell receptor
TGF-β: transforming growth factor beta
TIGIT: T-cell immunoreceptor tyrosine-based inhibition motif domain
TIL: tumor-infiltrating lymphocyte
TIM-3: T-cell immunoglobulin, mucin domain-3 protein
TLR3: toll-like receptor 3
TME: tumor microenvironment
TRUCK: T cells redirected for antigen‐unrestricted cytokine‐initiated killing
TVEC: talimogene laherparepvec
VEGF: vascular endothelial growth factor
VISTA: V-domain immunoglobulin-containing suppressor of T-cell activation
XCL1: XC-chemokine ligand 1
yCD: yeast cytosine deaminase
